# Transcatheter Aortic Valve Replacement for Severe Bicuspid Aortic Valve Stenosis: A Systematic Review and Meta-Analysis

**DOI:** 10.1016/j.jacadv.2026.103052

**Published:** 2026-08-26

**Authors:** Sant Kumar, Jung-Min Ahn, Euihong Ko, Gossadi Mohammed, Kentaro Hayashida, Wei-Hsian Yin, Hasan Jilaihawi, Raj Makkar, Won-Keun Kim, Tullio Palmerini, Christine J. Chung, David Elison, Soo Yeon An, Ju Hyeon Kim, Do-Yoon Kang, Joon Bum Kim, David J. Cohen, Davide Capodanno, Duk-Woo Park, Seung-Jung Park

**Affiliations:** a Department of Cardiology, Creighton University School of Medicine, Phoenix, Arizona, USA; b Division of Cardiology, University of Ulsan College of Medicine, Heart Institute, Asan Medical Center, Seoul, South Korea; c Department of Cardiology, Kokura Memorial Hospital, Kokura, Japan; d Consultant of Internal Medicine and Adult Cardiology, Ministry of Health, Saudi Arabia; e Department of cardiology, Keio University School of Medicine, Tokyo, Japan; f Division of Cardiology, Heart Center, Cheng Hsin General Hospital, Taipei, Taiwan; g Cedars-Sinai Medical Center Smidt Heart Institute, Los Angeles, California, USA; h Department of Cardiology, Kerckhoff Heart and Thorax Centre, Bad Nauheim, Germany; i IRCCS Azienda Ospedaliero-Universitaria di Bologna, Bologna, Italy; j Division of Cardiology, Department of Medicine, University of Washington, Seattle, Washington, USA; k Department of Thoracic and Cardiovascular Surgery, Asan Medical Center, University of Ulsan College of Medicine, Seoul, South Korea; l Cardiovascular Research Foundation, New York, New York, USA; m St. Francis Hospital, Roslyn, New York, USA; n Division of Cardiology, Azienda Ospedaliero-Universitaria Policlinico G. Rodolico - San Marco, University of Catania, Catania, Italy

**Keywords:** aortic valve stenosis, bicuspid aortic valve, meta-analysis, paravalvular leak, stroke, surgical aortic valve replacement, transcatheter aortic valve replacement

## Abstract

**Background:**

Comparative outcomes of transcatheter aortic valve replacement (TAVR) in bicuspid aortic valve (BAV) stenosis remain uncertain.

**Objectives:**

The authors synthesized evidence comparing: 1) TAVR outcomes in BAV vs tricuspid aortic valve (TAV) stenosis; and 2) TAVR vs surgical aortic valve replacement (SAVR) among patients with BAV stenosis.

**Methods:**

MEDLINE, Scopus, and Cochrane CENTRAL were searched through January 31, 2026 (CRD420261304542). Random-effects models pooled risk ratios (RRs) with 95% CIs. Exploratory meta-regression assessed study-level modifiers and temporal trends in 1-year mortality.

**Results:**

Fifty-two studies were included. In 39 studies comparing TAVR in BAV vs TAV, BAV was associated with higher paravalvular leak (RR: 1.44; 95% CI: 1.18-1.77; *P* < 0.001), aortic root injury (RR: 1.83; 95% CI: 1.10-3.04; *P* = 0.020), 30-day stroke (RR: 1.27; 95% CI: 1.02-1.59; *P* = 0.035), and 30-day mortality (RR: 1.25; 95% CI: 1.00-1.55; *P* = 0.047), but lower 1-year mortality (RR: 0.84; 95% CI: 0.75-0.94; *P* = 0.002). The Society of Thoracic Surgeons score—but not BAV anatomy—predicted 1-year mortality (RR: 1.31; 95% CI: 1.08-1.59; *P* = 0.006). BAV outcomes improved in recent studies (RR per year, 0.93; 95% CI: 0.87-1.00; *P* = 0.048). In 13 studies comparing TAVR vs SAVR in BAV, TAVR had higher paravalvular leak (RR: 7.08; 95% CI: 2.28-21.98; *P* < 0.001) and lower major bleeding (RR: 0.27; 95% CI: 0.17-0.44; *P* < 0.001) but similar 30-day stroke (*P* = 0.288) and mortality (*P* = 0.452).

**Conclusions:**

TAVR for BAV stenosis was associated with procedural risk than TAVR for TAV stenosis but lower 1-year mortality. Compared with SAVR, TAVR showed procedural trade-offs without early mortality differences.

Transcatheter aortic valve replacement (TAVR) has become an established therapy for severe symptomatic aortic stenosis (AS) across the surgical risk spectrum in patients with tricuspid aortic valve (TAV) anatomy.^1^ In contrast, bicuspid aortic valve (BAV) disease, present in 1% to 2% of the population and more common in younger patients requiring valve intervention, remains anatomically distinct and procedurally challenging.^2^ BAV is characterized by asymmetric leaflet fusion, calcified raphes, elliptical annular geometry, and frequent concomitant aortopathy.^2^ Because patients with BAV were excluded from pivotal randomized TAVR trials, surgical aortic valve replacement (SAVR) has traditionally been considered the standard therapy in this population. Additionally, direct head-to-head comparisons of TAVR outcomes in BAV by geographic region remain limited.

As TAVR expands into younger and lower-risk cohorts, where BAV prevalence is higher, defining the relative safety and efficacy of TAVR in BAV anatomy has become increasingly important. Contemporary observational data suggest comparable outcomes in selected patients, particularly with newer-generation devices; however, concerns persist regarding paravalvular leak (PVL), permanent pacemaker implantation (PPM), aortic root injury, and stroke.^3^, ^4^, ^5^ Prior meta-analyses have been limited by heterogeneous endpoint definitions and limited contemporary representation.^6^, ^7^, ^8^

Accordingly, we conducted a comprehensive and contemporary systematic review and meta-analysis to evaluate procedural, short-term, and available 1-year clinical outcomes of TAVR in BAV. We compared 1) TAVR in BAV vs TAV stenosis and 2) TAVR vs SAVR in BAV stenosis to better define early risks and clarify the evolving role of TAVR in this anatomically complex population.

## Methods

### Study design and eligibility criteria

This meta-analysis was conducted in accordance with the Preferred Reporting Items for Systematic Reviews and Meta-Analyses statement and was prospectively registered in the International Prospective of Register Systematic Reviews (PROSPERO; CRD420261304542). The study was designed according to a predefined PICOS (Population, Interventions, Outcomes, and Study) framework. The population included adult patients with severe AS and BAV anatomy. The interventions were TAVR and SAVR. The comparisons were: 1) TAVR in BAV vs TAVR in TAV stenosis; and 2) TAVR vs SAVR in patients with BAV stenosis. The outcomes included significant PVL defined as PVL with moderate or severe grade, PPM, aortic root injury (annular rupture and/or aortic dissection), major bleeding, 30-day mortality, 30-day stroke, 1-year mortality, and 1-year stroke. Outcomes were pooled using event-level data as reported in the individual studies. Eligible study designs included randomized controlled trials, prospective registries, and retrospective cohort studies.

Studies were eligible if they enrolled adult patients with severe BAV AS and reported comparative outcomes for either TAVR in BAV vs TAV stenosis or TAVR vs SAVR in BAV stenosis. Studies were excluded if they were meta-analyses/systematic reviews, case reports, small case series (<10 patients), single-arm analyses without a comparator group, or did not provide extractable outcome data. When multiple reports described overlapping populations, the most comprehensive or contemporary data set was included.

### Search strategy and data collection

A comprehensive search of MEDLINE (via PubMed), Scopus, and Cochrane CENTRAL was performed from database inception through January 2026. The full electronic search strategy for each database is detailed in Supplemental Table 1. Search terms combined controlled vocabulary and free-text terms related to BAV, AS, TAVR/transcatheter aortic valve implantation, and SAVR. Reference lists of eligible studies were manually screened to identify additional relevant publications. No language restrictions were applied.

Two investigators (S.K. and J.M.A.) independently screened titles and abstracts, followed by full-text review of potentially eligible studies. Disagreements were resolved by consensus. Two reviewers independently extracted data using a standardized data collection form, and extracted data were cross-verified for accuracy. Extracted variables included study characteristics (author, year, and design), sample size, baseline demographics (age and sex), operative risk when reported, valve platform, and clinical outcomes. Because only published, study-level data were analyzed, this meta-analysis was exempt from Institutional Review Board oversight.

Risk of bias was assessed using a modified Newcastle–Ottawa Scale, evaluating cohort representativeness, comparability at baseline, outcome assessment, and adequacy of follow-up (Supplemental Table 2). Publication bias was evaluated using funnel plot inspection and Egger’s regression testing. Forest plots and publication bias assessments are presented in Supplemental Figures 1 and 2.

### Statistical analysis

Statistical analyses were performed using random-effects models. Meta-analyses were conducted using the Mantel-Haenszel method to account for between-study heterogeneity. Dichotomous outcomes were pooled as risk ratios (RRs) with 95% CIs, and continuous outcomes were pooled as mean differences (MDs). Because only aggregate study-level event counts were available, outcomes were analyzed separately as reported, and competing-risk models could not be applied. Moreover, outcomes were extracted as cumulative event counts at the reported time points. Because censoring information, person-time, and numbers at risk were not consistently available across studies, censoring-adjusted time-to-event models were not performed. Follow-up outcomes were therefore analyzed as reported binary event risks within each time window.

Zero-event studies were handled on an outcome-specific basis. For studies with zero events in one comparison arm, a standard continuity correction of 0.5 was applied to all cells of the corresponding 2×2 table to allow estimation of the RR and its variance. Studies with zero events in both arms for a given outcome were retained in the descriptive tables but were not assigned weight in the pooled RR for that endpoint because the study-specific relative effect was not estimable. Outcomes that were not reported were treated as missing and excluded only from the corresponding endpoint-specific analysis.

Overall pooled analyses were prespecified as the main analyses. When both crude and propensity-matched estimates were available from the same study, the propensity-matched estimate was used in the overall analysis. Crude and propensity-matched cohorts were additionally analyzed as subgroups to assess robustness and formally test for subgroup differences. Statistical heterogeneity was assessed using the Cochran Q test and quantified using the I^2^ statistic, with I^2^ >50% considered indicative of moderate-to-substantial heterogeneity. When necessary, means and SDs were estimated from medians and IQRs using validated conversion methods.^9^^,^^10^

Exploratory study-level meta-regression analyses were performed for 30-day mortality, 30-day stroke, 1-year mortality, and 1-year stroke. Univariable models evaluated BAV vs TAV status, valve-platform (self-expanding valve vs balloon-expandable valve), the Society of Thoracic Surgeons (STS) risk score, male sex, and age. Multivariable models then included these covariates simultaneously to explore whether study-level differences in case mix or valve platform were associated with between-study variation in effect estimates.

An exploratory era-based subgroup analysis was conducted for the outcome of 1-year mortality, stratifying studies by publication period (2013-2018 vs 2019-2025) to evaluate temporal trends in outcomes between bicuspid and TAV stenosis. Subgroup pooled RRs were calculated using the same random-effects model, and subgroup interactions were assessed. Additionally, inverse-variance weighted meta-regression was performed to evaluate whether the relative risk of 1-year mortality between BAV and TAV changed over time. The natural logarithm of the RR (log RR) was regressed against publication year. Regression coefficients were exponentiated to express the change in RR per year of publication.

Statistical analyses were performed using Review Manager (RevMan), version 5.4 (Cochrane Collaboration, 2020). A 2-sided *P* value <0.05 was considered statistically significant.

## Results

**Figure 1:**
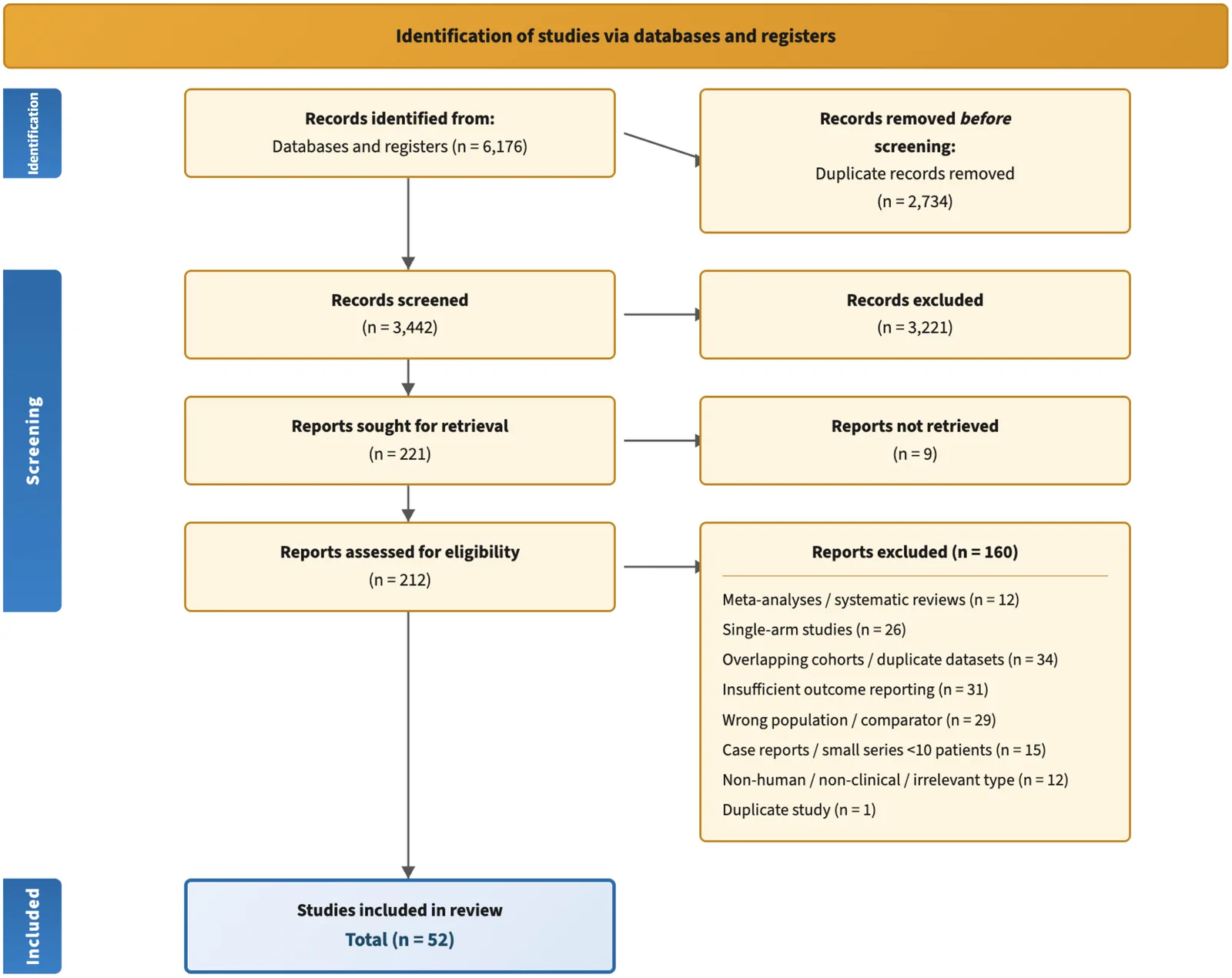
**Preferred Reporting Items for Systematic Reviews and Meta-Analyses Flow Diagram** Flow diagram illustrating study identification, screening, eligibility assessment, and inclusion in the meta-analysis.

Fifty-two studies were included in the final review (Figure 1).^3^^,^^11^, ^12^, ^13^, ^14^, ^15^, ^16^, ^17^, ^18^, ^19^, ^20^, ^21^, ^22^, ^23^, ^24^, ^25^, ^26^, ^27^, ^28^, ^29^, ^30^^,^^31^, ^32^, ^33^, ^34^, ^35^, ^36^, ^37^, ^38^, ^39^, ^40^, ^41^, ^42^, ^43^, ^44^, ^45^, ^46^, ^47^, ^48^, ^49^, ^50^, ^51^, ^52^, ^53^, ^54^, ^55^, ^56^, ^57^, ^58^, ^59^, ^60^, ^61^ Among studies comparing TAVR in BAV vs TAV stenosis, 39 studies contributed data overall (Table 1).^3^^,^^11^, ^12^, ^13^, ^14^, ^15^, ^16^, ^17^, ^18^, ^19^, ^20^, ^21^, ^22^, ^23^, ^24^, ^25^, ^26^, ^27^, ^28^, ^29^, ^30^, ^31^, ^32^, ^33^, ^34^, ^35^, ^36^, ^37^, ^38^, ^39^, ^40^, ^41^, ^42^, ^43^, ^44^, ^45^, ^46^, ^47^, ^48^ Baseline characteristics are summarized in Supplemental Table 3. Patients with BAV were significantly younger than those with TAV (MD −2.77 years; 95% CI: -3.70 to −1.84; *P* < 0.001) (Supplemental Figure 3), while the proportion of male patients was similar between groups (RR: 1.14; 95% CI: 0.97-1.33; *P* = 0.102) (Supplemental Figure 4). The primary results are from the overall pooled analyses, in which propensity-matched estimates were used when both crude and matched data were available; crude and propensity-matched subgroup results are presented separately in Supplemental Tables 4 and 5.

**Table 1:** Studies for Comparative Outcomes of Transcatheter Aortic Valve Replacement in Bicuspid versus Tricuspid Aortic Valve Stenosis

First Author	Year	Number		Paravalvular Leak ≥ Moderate		Permanent Pacemaker Implantation		Aortic Root Injury^a^		Major Bleeding		30-Day Mortality		30-Day Stroke		1-Year Mortality		1-Year Stroke	
		BAV	TAV	BAV	TAV	BAV	TAV	BAV	TAV	BAV	TAV	BAV	TAV	BAV	TAV	BAV	TAV	BAV	TAV
Hayashida	2013	21	208	0 (0%)	2 (1.0%)	3 (14.3%)	15 (7.2%)	0 (0%)	3 (1.4%)	1 (4.8%)	9 (4.3%)	1 (4.8%)	17 (8.2%)	NR	NR	NR	NR	NR	NR
Bauer	2014	38	1,357	10 (25%)	204 (15%)	6 (17%)	475 (35%)	1 (2.6%)	5 (0.4%)	NR	NR	4 (11%)	149 (11%)	0 (0%)	41 (3%)	NR	NR	NR	NR
Costopoulos	2014	21	447	0 (0%)	11 (3%)	3 (14%)	67 (15%)	NR	NR	4 (19%)	90 (20%)	3 (16%)	16 (3.6%)	0 (0%)	5 (1.0%)	6 (32%)	52 (14%)	NR	NR
Kochman	2014	28	84	9 (32%)^c^	19 (23%)^c^	8 (28%)	28 (33%)	0 (0%)	0 (0%)	3 (11%)^b^	12 (14%)^b^	1 (4%)	6 (7%)	0 (0%)	3 (4%)	5/23 (18%)	14/70 (17%)	NR	NR
Liu	2015	15	25	0 (0%)	1 (4.0%)	2 (13.3%)	3 (12.0%)	0 (0%)	0 (0%)	1 (6.7%)	5 (20.0%)	1 (6.7%)	2 (8.0%)	1 (6.7%)	1 (4.0%)	NR	NR	NR	NR
Watanabe	2015	11	56	5 (45.5%)^c^	15 (26.8%)^c^	NR	NR	0 (0%)	2 (11.1%)	NR	NR	NR	NR	NR	NR	NR	NR	NR	NR
Arai	2017	10	143	0 (0%)	8 (6%)^c^	0 (0%)	12 (8%)	0 (0%)	1 (1%)	0 (0%)	1 (1%)^b^	0 (0%)	1 (1%)	0 (0%)	0 (0%)	NR	NR	NR	NR
Sannino	2017	88	735	4 (5.3%)	32 (5.0%)	20 (22.7%)	133 (18.2%)	NR	NR	3 (3.4%)	15 (2.0%)	3 (3.4%)	23 (3.1%)	2 (2.3%)	27 (3.7%)	7 (8.5%)	68 (10.5%)	NR	NR
Yoon	2017	546^d^	546^d^	57 (10.4%)	37 (6.8%)	84 (15.4%)	84 (15.4%)	9 (1.6%)	0 (0%)	20 (3.7%)	22 (4.0%)	20 (3.7%)	18 (3.3%)	16 (2.9%)	10 (1.8%)	66 (12.1%)	73 (13.4%)	NR	NR
Liao	2018	87	70	1 (1.1%)	0 (0%)	21 (24.1%)	20 (28.6%)	0 (0%)	0 (0%)	12 (13.8%)	7 (10%)	8 (9.2%)	3 (4.3%)	1 (1.1%)	0 (0%)	NR	NR	NR	NR
Aalaei-Andabili	2018	32	96	1 (3.1%)	2 (2.1%)	4 (12.5%)	13 (13.5%)	NR	NR	NR	NR	2 (6.3%)	4 (4.2%)	2 (6.3%)	3 (3.1%)	5 (15.6%)	10 (10.4%)	NR	NR
De Biase	2018	83^d^	166^d^	3 (3%)	4 (2%)	12 (14%)	17 (10%)	1 (1.2%)	0 (0%)	NR	NR	4 (5%)	5 (3%)	0 (0%)	1 (0.6%)	NR	NR	NR	NR
Kim	2018	144	288	16 (11.1%)	8 (2.8%)	*NR*	NR	6 (4.2%)	2 (0.7%)	NR	NR	7 (4.9%)	10 (3.5%)	NR	NR	26 (18.2%)	57 (19.8%)	NR	NR
Nagaraja	2018	359^d^	359^d^	NR	NR	40 (11.1%)	15 (4.2%)	NR	NR	64 (17.8%)	85 (23.7%)	20 (5.6%)	5 (1.4%)	10 (2.8%)	20 (5.6%)	NR	NR	NR	NR
Xiong	2018	67	49	4 (6.3%)	3 (6.3%)	17 (25.4%)	11 (22.4%)	NR	NR	NR	NR	6 (9.0%)	2 (4.1%)	NR	NR	6 (9.0%)	5 (10.2%)	1 (1.5%)	1 (2.0%)
Mangieri	2018	54	658	4 (7.4%)	9 (3.1%)	5 (9.2%)	57 (8.6%)	0 (0%)	0 (0%)	2 (3.7%)	34 (5.1%)	2 (3.7%)	17 (2.8%)	4 (7.4%)	12 (1.8%)	NR	NR	NR	NR
Tchetche	2019	101	88	21 (20.8%)^c^	11 (12.5%)^c^	13 (13%)	12 (14%)	NR	NR	11 (11%)	4 (4.5%)	0 (0%)	3 (3.4%)	2 (2%)	0 (0%)	NR	NR	NR	NR
Pineda	2020	50	517	2 (4.0%)	19 (3.7%)	4 (8%)	73 (14.1%)	1 (2%)	3 (0.6%)	1 (2.0%)	1 (0.2%)	3 (6.0%)	8 (1.5%)	NR	NR	8 (16.0%)	54 (10.4%)	NR	NR
Forrest	2020	929^d^	929^d^	47 (5.6%)	18 (2.1%)	133 (14.3%)	115 (12.4%)	NR	NR	1 (0.1%)	1 (0.1%)	23 (2.6%)	15 (1.7%)	31 (3.4%)	25 (2.7%)	62 (10.4%)	69 (12.4%)	33 (3.9%)	34 (4.4%)
Halim	2020	5,412	165,547	215/4,856 (4.4%)	4,753/149,442 (3.2%)	NR	NR	NR	NR	303/5,335 (5.7%)	10,042/163,278 (6.2%)	NR	NR	NR	NR	NR	NR	NR	NR
Kim	2021	242^d^	726^d^	5 (2.1%)	17 (2.3%)	38 (15.7%)	91 (12.5%)	3 (1.2%)	5 (0.7%)	17 (7.0%)	51 (7.0%)	4 (1.7%)	19 (2.6%)	9 (3.7%)	21 (2.9%)	NR	NR	*NR*	NR
Makkar	2021	3,168^d^	3,168^d^	38 (1.8%)	24 (1.1%)	189 (6.2%)	156 (5.2%)	12 (0.4%)	9 (0.3%)	0 (0%)	0 (0%)	28 (0.9%)	24 (0.8%)	42 (1.4%)	37 (1.2%)	70 (4.6%)	101 (6.6%)	49 (2.0%)	49 (2.0%)
Medranda	2021	47	60	0 (0%)	1 (1.7%)	3 (6.4%)	1 (1.7%)	NR	NR	NR	NR	0 (0%)	0 (0%)	1 (2.1%)	0 (0%)	0 (0%)	1/59 (1.7%)	2/43 (4.7%)	1/58 (1.7%)
Xu	2021	9	14	0 (0%)	0 (0%)	NR	NR	NR	NR	NR	NR	0 (0%)	0 (0%)	0 (0%)	0 (0%)	0 (0%)	1 (7.1%)	NR	NR
Jung	2021	19	48	NR	NR	0 (0%)	2 (4.2%)	0 (0%)	0 (0%)	0 (0%)	1 (2.1%)	NR	NR	NR	NR	NR	NR	NR	NR
Deeb	2022	145^d^	145^d^	2 (1.4%)	3 (2.1%)	21 (14.9%)	25 (17.2%)	NR	NR	2 (1.4%)	4 (2.8%)	1 (0.7%)	0 (0%)	6 (4.1%)	3 (2.1%)	1 (0.7%)	3 (2.1%)	7 (4.8%)	4 (2.8%)
Williams	2022	148^d^	148^d^	NR	NR	9 (6.1%)	10 (6.8%)	NR	NR	NR	NR	0 (0%)	0 (0%)	2 (1.4%)	2 (1.4%)	1 (0.7%)	2 (1.4%)	3 (2.0%)	3 (2.0%)
Fan	2022	223	172	NR	NR	20 (9.0%)	14 (8.1%)	6 (2.7%)	4 (2.3%)	NR	NR	7 (3.3%)	1 (0.6%)	7 (3.3%)	5 (3.1%)	NR	NR	NR	NR
Jin	2022	195	149	27 (13.8%)	10 (6.7%)	25 (12.8%)	22 (14.8%)	0 (0%)	1 (0.7%)	2 (1.0%)	2 (1.3%)	3 (1.5%)	4 (2.7%)	10 (5.1%)	3 (2.0%)	7 (3.6%)	8 (5.37%)	12 (6.2%)	5 (3.4%)
Hong	2023	229	160	6 (2.6%)	4 (2.5%)	15 (6.6%)	14 (8.8%)	NR	NR	6 (2.6%)	2 (1.3%)	2 (0.9%)	1 (0.7%)	7 (3.3%)	0 (0%)	NR	NR	NR	NR
Mukai	2023	423	16,802	13 (3.2%)	290 (1.7)	19 (4.5)	880 (5.2)	0 (0)	46 (0.3%)	1 (0.2%)	54 (0.3%)	2 (0.5)	214 (1.3)	8 (1.9)	213 (1.3)	34 (7.9%)	1,361 (8.1%)	NR	NR
Zheng	2023	185	482	6 (6.7%)	13 (6.5%)	8 (8.9%)	20 (10.0%)	NR	NR	NR	NR	NR	NR	NR	NR	NR	NR	NR	NR
Liu	2024	29	36	2 (6.9%)	2 (5.6%)	1 (3.4%)	5 (13.9%)	0 (0%)	0 (0%)	0 (0%)	0 (0%)	NR	NR	NR	NR	NR	NR	NR	NR
Li	2024	170	145	3 (1.8%)	0 (0%)	43 (25.7%)	34 (24.8%)	NR	NR	17 (10.0%)	6 (4.2%)	NR	NR	NR	NR	NR	NR	NR	NR
Yamanaka	2025	497^d^	497^d^	11 (2.2%)	18 (3.6%)	38 (7.7%)	44 (8.9%)	0 (0%)	2 (2.0%)	15 (3.0%)	36 (7.2%)	5 (1.0%)	5 (1.0%)	NR	NR	NR	NR	NR	NR
Zhu	2025	508	646	43 (8.5%)	86 (13.3%)	67 (13.2%)	67 (10.4%)	NR	NR	NR	NR	NR	NR	NR	NR	NR	NR	NR	NR
Sheng	2025	193	234	4 (2.1%)	6 (2.6%)	9 (4.7%)	28 (12.0%)	0 (0%)	0 (0%)	21 (10.9%)	28 (12.0%)	NR	NR	NR	NR	9 (4.7%)	21 (9.0%)	5 (2.6%)	4 (1.7%)
Louca	2025	577^d^	2,885^d^	25 (4.3%)	78 (2.7%)	67 (12%)	188 (6.9%)	NR	NR	NR	NR	10 (1.7%)	58 (2.0%)	NR	NR	NR	NR	NR	NR
Sun	2025	870^d^	870^d^	32/722 (4.4%)	32/735 (4.4%)	61 (7.0%)	45 (5.2%)	3 (0.3%)	3 (0.3%)	10 (1.1%)	3 (0.3%)	NR	NR	NR	NR	38 (4.3%)	46 (5.3%)	13 (1.5%)	12 (1.4%)
39 studies		15,973	202, 201	616/14,520 (4.2%)	5,740/182,788 (3.1%)	1,008/10,397 (9.7%)	2,796/33,850 (8.3%)	36/6,723 (0.5%)	82/26,648 (0.3%)	517/13,824 (3.7%)	10,516/191,067 (5.5%)	169/8,576 (2.0%)	636/31,691 (2.0%)	161/7,220 (2.2%)	432/27,247 (1.6%)	351/7,098 (4.9%)	2,004/25,266 (7.9%)	125/5,758 (2.2%)	113/5,701 (2.0%)
				1.44 (95% CI 1.18-1.77) *P* < 0.001		1.08 (95% CI 0.94-1.24) *P* = 0.276		1.83 (95% CI 1.10-3.04) *P* = 0.020		0.96 (95% CI 0.77-1.20) *P* = 0.718		1.25 (95% CI 1.00-1.55) *P* = 0.046		1.27 (95% CI 1.02-1.59) *P* = 0.035		0.84 (95% CI 0.75-0.94) *P* = 0.002		1.00 (95% CI 0.83-1.20) *P* = 1.00	

BAV = bicuspid aortic valve; NR = not reported; TAV = tricuspid aortic valve.

a Aortic dissection and/or annulus rupture.

b Lethal bleeding.

c ≥ Mild.

d Propensity-matched population.

**Figure 2:**
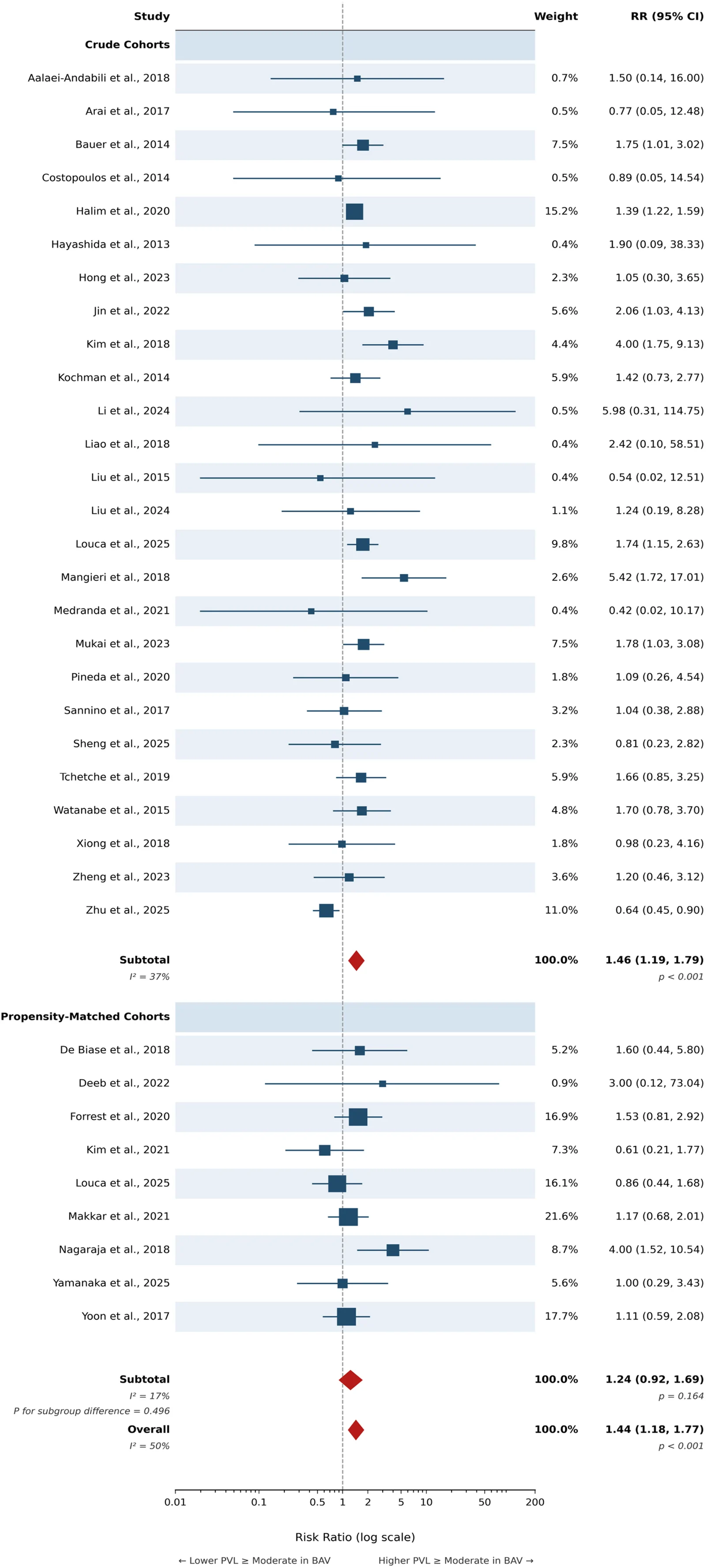
**Forest Plot for Moderate or Greater Paravalvular Leak (≥ Moderate) in Transcatheter Aortic Valve Replacement for Bicuspid vs Tricuspid Aortic Valve Stenosis** Forest plot comparing the risk of moderate or greater paravalvular leak after transcatheter aortic valve replacement in bicuspid vs tricuspid aortic valve stenosis. BAV = bicuspid aortic valve; PVL = paravalvular leak; RR = risk ratio.

**Figure 3:**
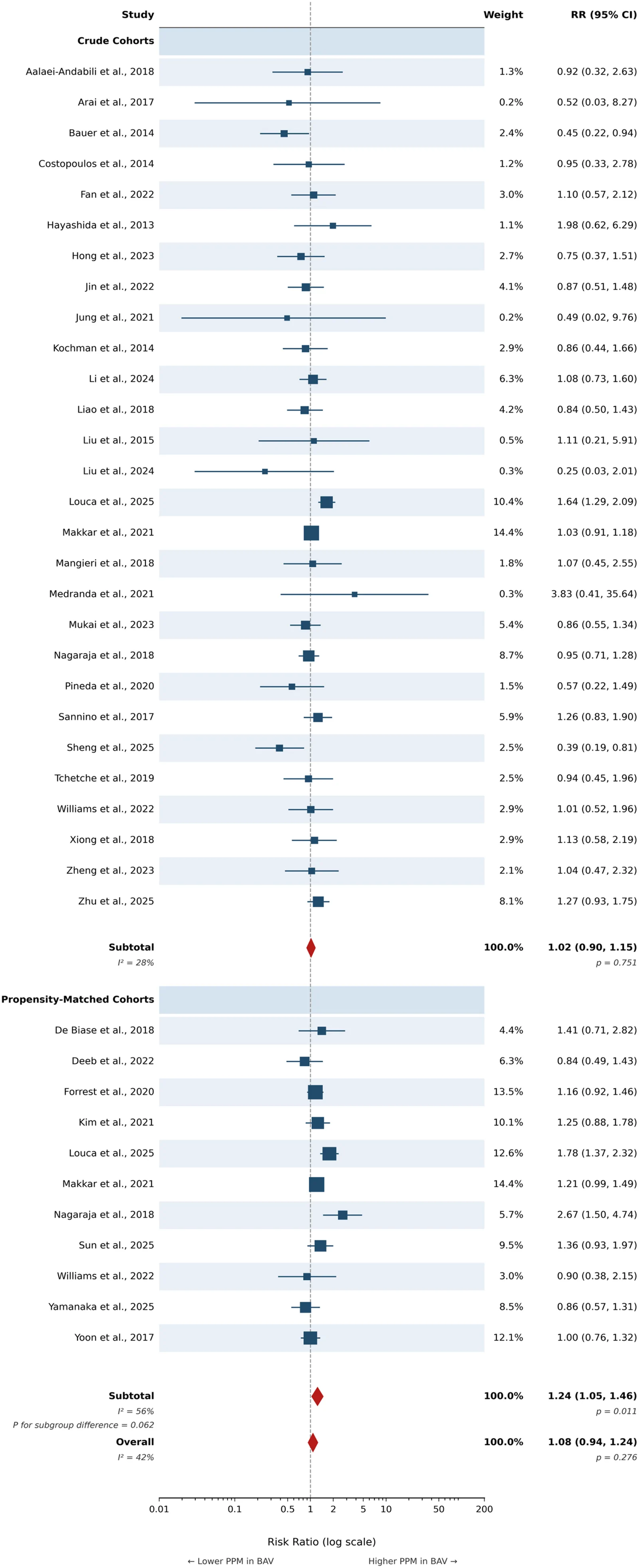
**Forest Plot for Permanent Pacemaker Implantation in Transcatheter Aortic Valve Replacement for Bicuspid vs Tricuspid Aortic Valve Stenosis** Forest plot comparing rates of permanent pacemaker implantation after transcatheter aortic valve replacement in bicuspid vs tricuspid aortic valve stenosis. PPM = permanent pacemaker implantation; other abbreviations as in Figure 2.

**Figure 4:**
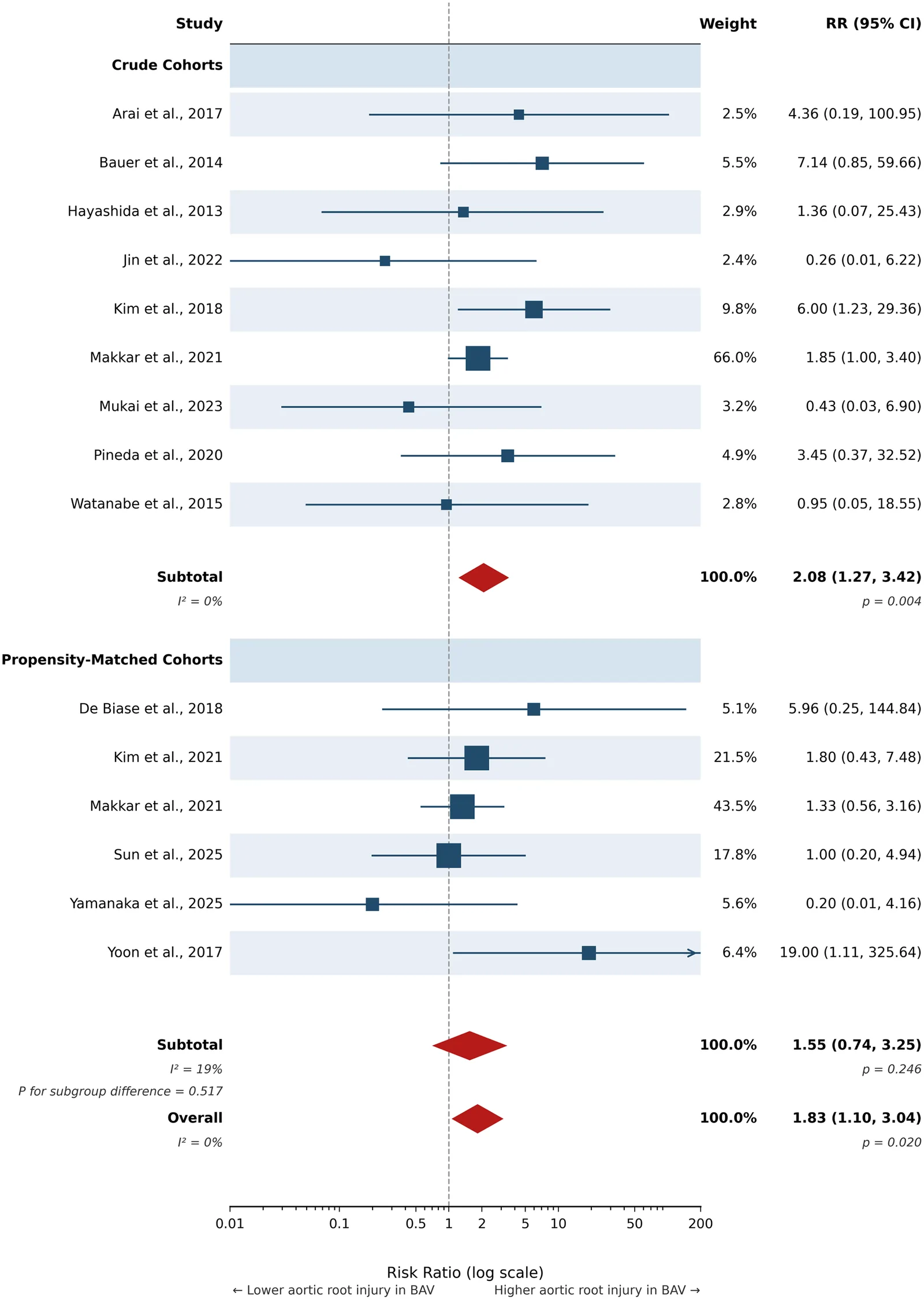
**Forest Plot for Aortic Root Injury in Transcatheter Aortic Valve Replacement for Bicuspid vs Tricuspid Aortic Valve Stenosis** Forest plot comparing the risk of aortic root injury after transcatheter aortic valve replacement in bicuspid vs tricuspid aortic valve stenosis. Abbreviations as in Figure 2.

**Figure 5:**
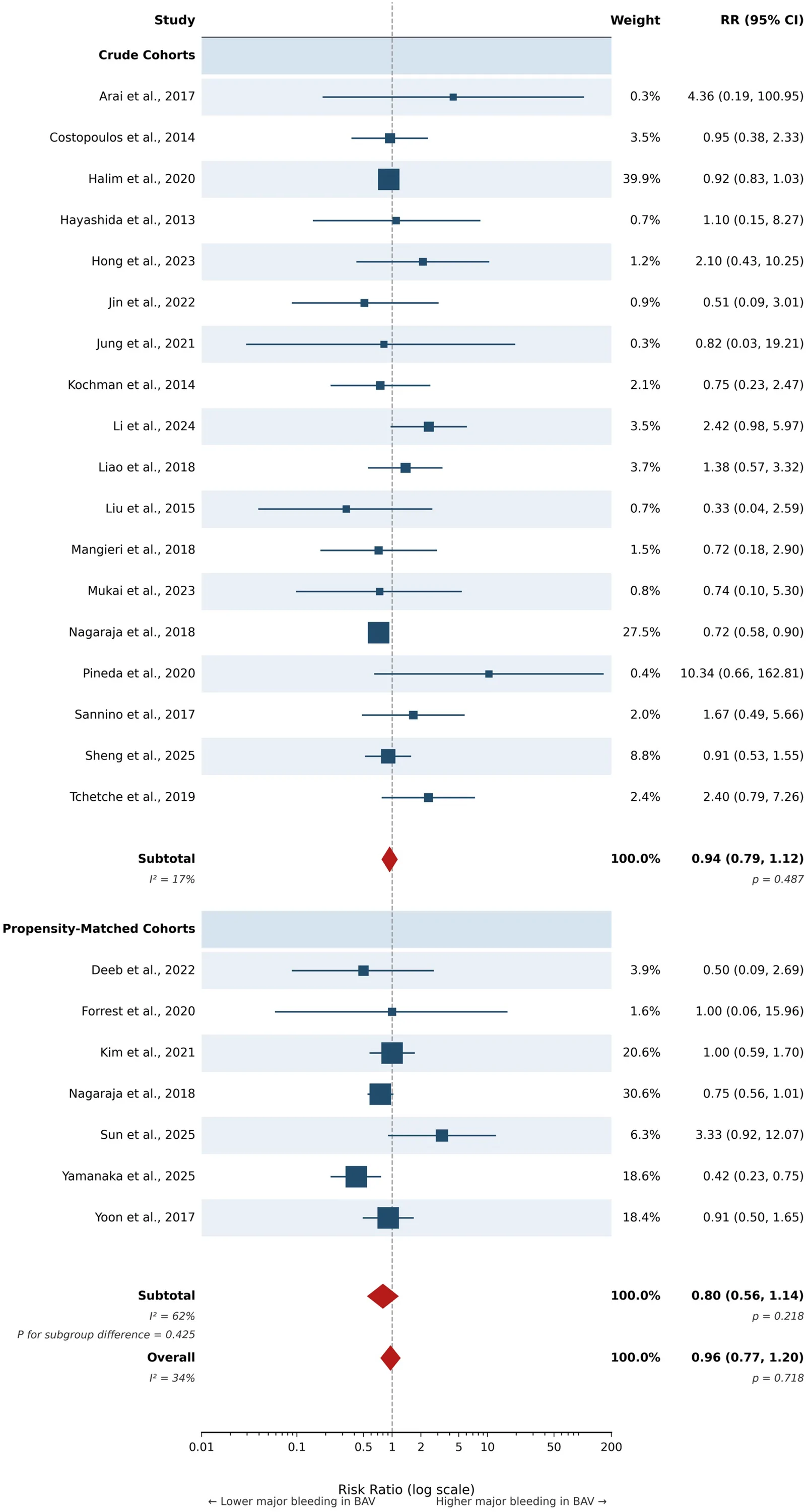
**Forest Plot for Major Bleeding in Transcatheter Aortic Valve Replacement for Bicuspid vs Tricuspid Aortic Valve Stenosis** Forest plot comparing rates of major bleeding after transcatheter aortic valve replacement in bicuspid vs tricuspid aortic valve stenosis. Abbreviations as in Figure 2.

**Figure 6:**
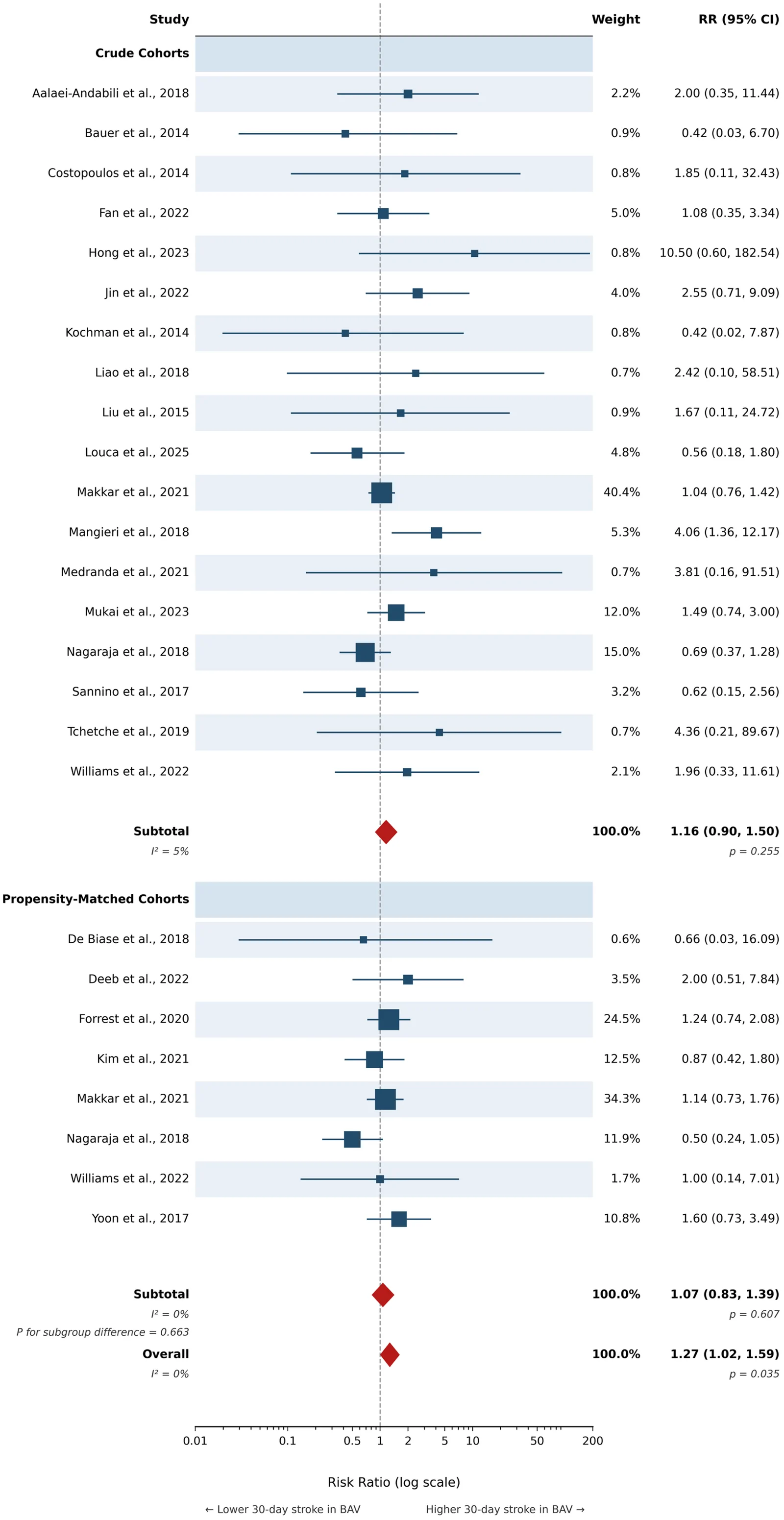
**Forest Plot for 30-Day Stroke in Transcatheter Aortic Valve Replacement for Bicuspid vs Tricuspid Aortic Valve Stenosis** Forest plot comparing 30-day stroke after transcatheter aortic valve replacement in bicuspid vs tricuspid aortic valve stenosis. Abbreviations as in Figure 2.

**Figure 7:**
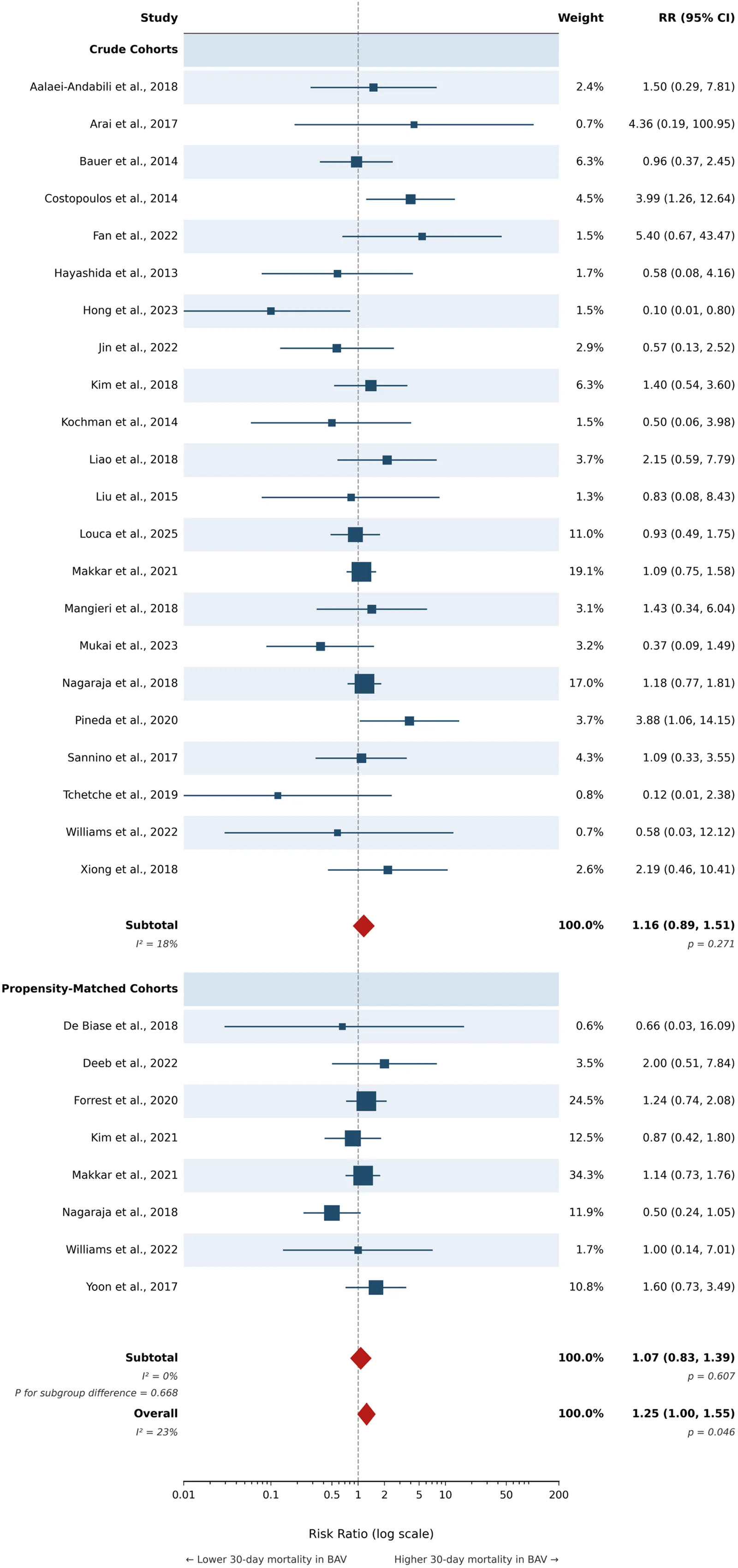
**Forest Plot for 30-Day Mortality in Transcatheter Aortic Valve Replacement for Bicuspid vs Tricuspid Aortic Valve Stenosis** Forest plot comparing 30-day mortality after transcatheter aortic valve replacement in bicuspid vs tricuspid aortic valve stenosis. Abbreviations as in Figure 2.

**Figure 8:**
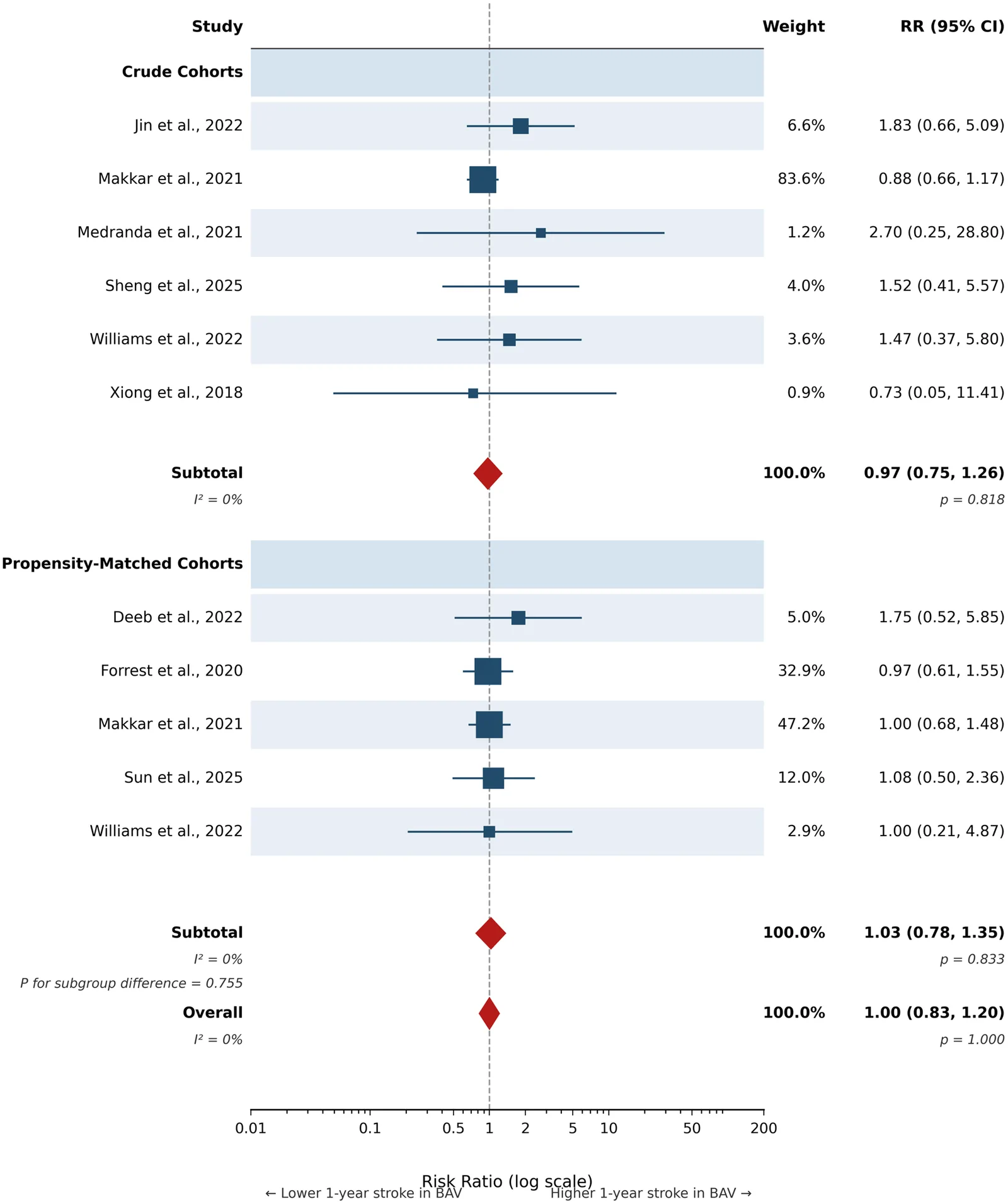
**Forest Plot for 1-Year Stroke in Transcatheter Aortic Valve Replacement for Bicuspid Aortic Valve vs Tricuspid Aortic Valve Aortic Stenosis** Forest plot comparing 1-year stroke after transcatheter aortic valve replacement in bicuspid vs tricuspid aortic valve stenosis. Abbreviations as in Figure 2.

**Figure 9:**
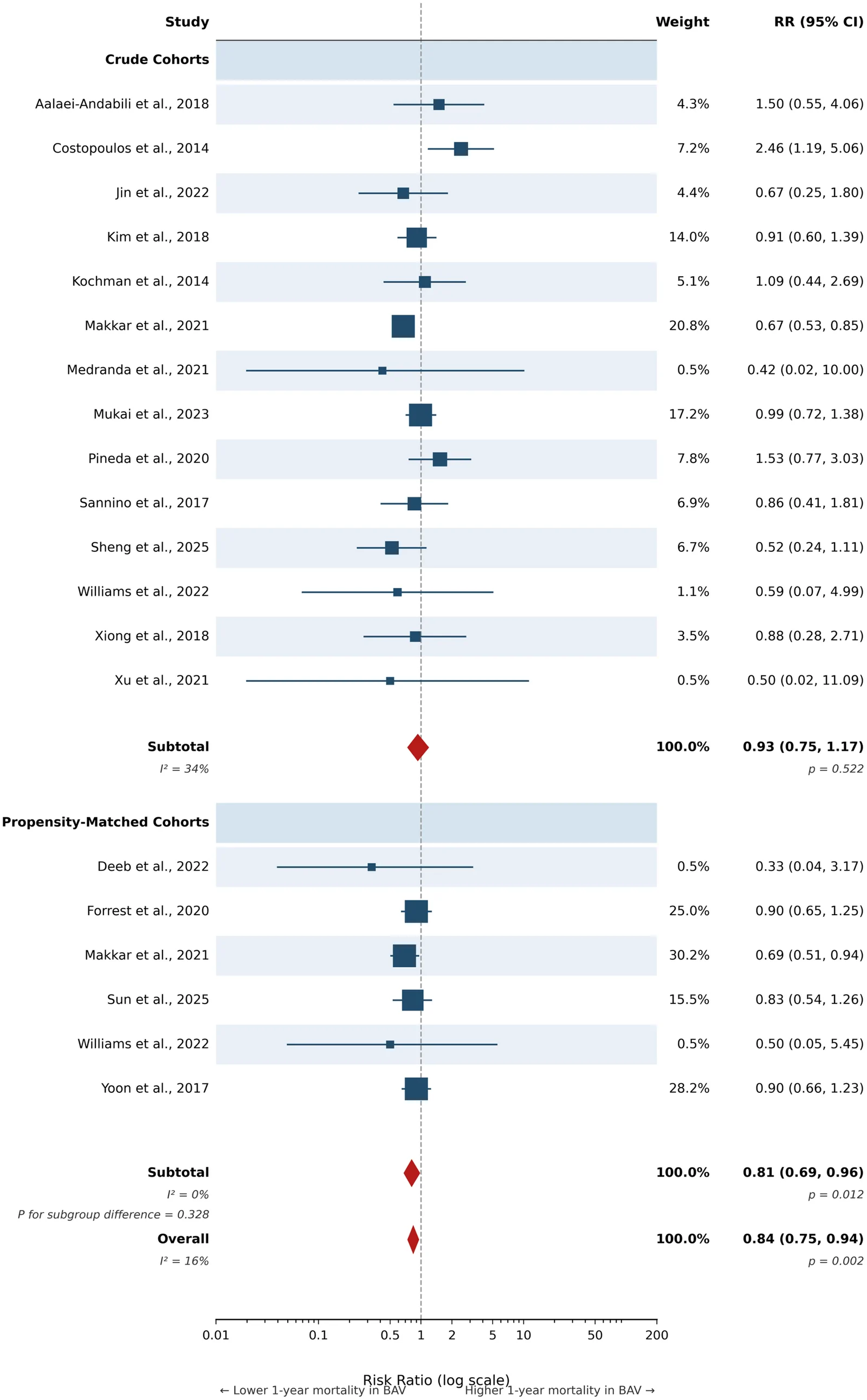
**Forest Plot for 1-Year Mortality in Transcatheter Aortic Valve Replacement for Bicuspid Aortic Valve vs Tricuspid Aortic Valve Aortic Stenosis** Forest plot comparing 1-year mortality after transcatheter aortic valve replacement in bicuspid vs tricuspid aortic valve stenosis. Abbreviations as in Figure 2.

In overall pooled analyses, moderate or greater PVL occurred more frequently in BAV than in TAV (RR: 1.44; 95% CI: 1.18-1.77; *P* < 0.001) (Figure 2). PPM did not differ significantly between groups (RR: 1.08; 95% CI: 0.94-1.24; *P* = 0.276) (Figure 3). Aortic root injury was more frequent in BAV (RR: 1.83; 95% CI: 1.10-3.04; *P* = 0.020) (Figure 4), whereas major bleeding was similar between groups (RR: 0.96; 95% CI: 0.77-1.20; *P* = 0.718) (Figure 5). BAV was also associated with higher 30-day stroke (RR: 1.27; 95% CI: 1.02-1.59; *P* = 0.035) (Figure 6) and higher 30-day mortality (RR: 1.25; 95% CI: 1.00-1.55; *P* = 0.047) (Figure 7). However, at 1 year, stroke remained similar (RR: 1.00; 95% CI: 0.83-1.20; *P* = 0.999) (Figure 8), whereas 1-year mortality was lower in BAV (RR: 0.84; 95% CI: 0.75-0.94; *P* = 0.002) (Figure 9).

Because crude and propensity-matched studies may estimate different underlying constructs, we examined crude and propensity-matched cohorts separately as sensitivity analyses within each applicable forest plot (Figures 2, 3, 4, 5, 6, 7, 8, and 9). Overall, crude and propensity-matched estimates were generally concordant in direction. In the BAV vs TAV comparison, the only directionally discordant result was 1-year stroke, for which the crude estimate was slightly below the null, RR: 0.97; 95% CI: 0.75-1.26; *P* = 0.818, and the propensity-matched estimate was slightly above the null, RR: 1.03; 95% CI: 0.78-1.35; *P* = 0.833; both were nonsignificant. Other differences were primarily in magnitude or statistical significance rather than direction, including PPM, aortic root injury, and 1-year mortality. In the TAVR vs SAVR comparison, crude and propensity-matched estimates were directionally consistent across outcomes, including higher PPM with TAVR and lower major bleeding with TAVR.

**Figure 10:**
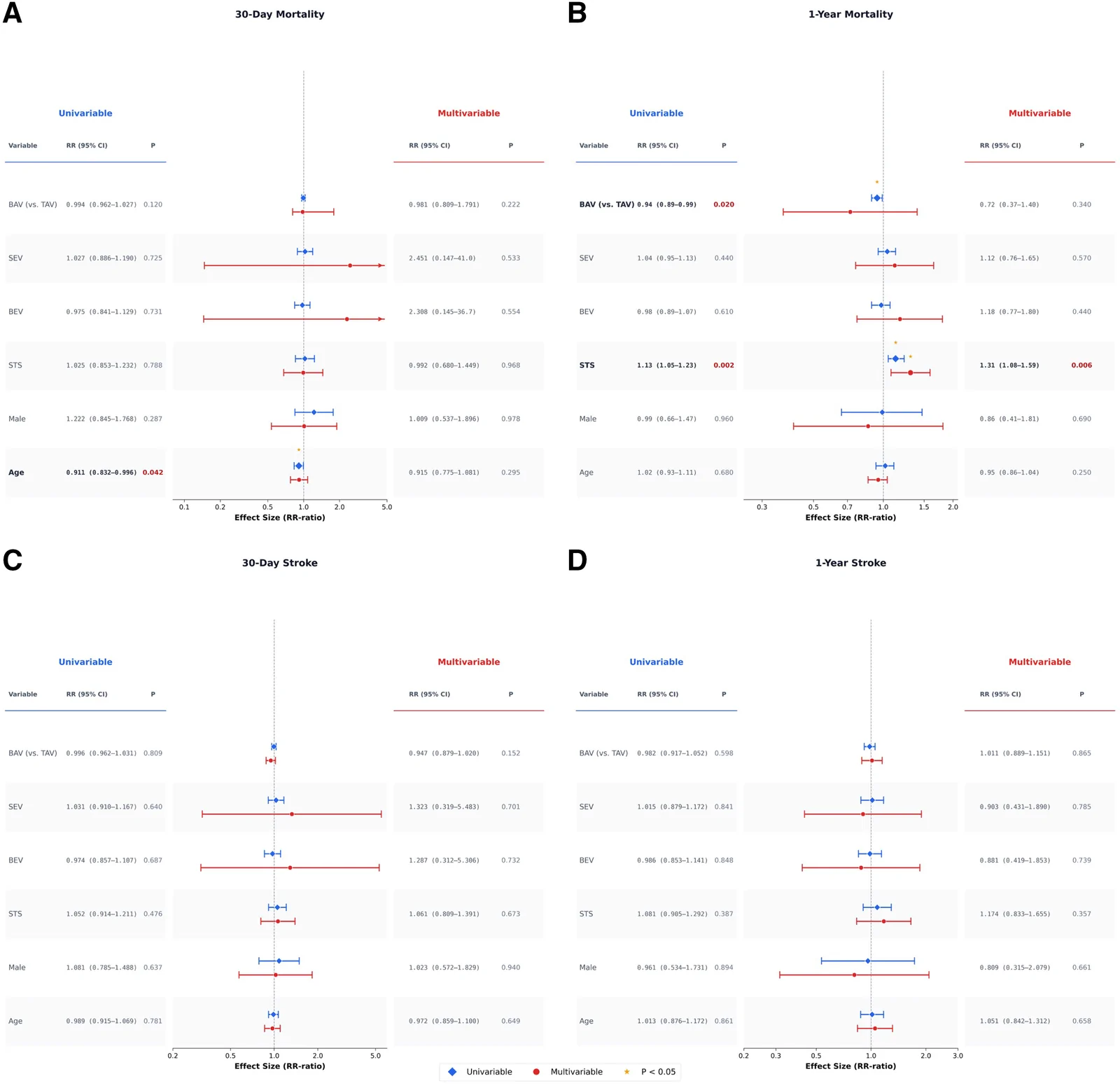
**Meta-regression of Transcatheter Aortic Valve Replacement in Bicuspid vs Tricuspid Aortic Valve Stenosis** Meta-regression evaluating the association between study-level covariates and outcomes of transcatheter aortic valve replacement in bicuspid vs tricuspid aortic valve stenosis: (A) 30-day mortality, (B) 1-year mortality, (C) 30-day stroke, and (D) 1-year stroke. BEV = balloon-expandable valve; SEV = self-expanding valve; STS = Society of Thoracic Surgeons; TAV = tricuspid aortic valve; other abbreviations as in Figure 2.

In exploratory meta-regression, no study-level variable remained significantly associated with 30-day mortality after multivariable adjustment; in univariable analysis, younger age was associated with lower 30-day mortality (RR: 0.911; 95% CI: 0.832-0.996; *P* = 0.042), but this signal was attenuated in the multivariable model. For 1-year mortality, BAV vs TAV status was associated with lower risk in univariable analysis (RR: 0.94; 95% CI: 0.89-0.99; *P* = 0.020), whereas higher STS score was associated with higher risk in both univariable (RR: 1.13; 95% CI: 1.05-1.23; *P* = 0.002) and multivariable models (RR: 1.31; 95% CI: 1.08-1.59; *P* = 0.006). Meta-regression analyses for 30-day stroke and 1-year stroke did not identify significant univariable or multivariable moderators. These meta-regression findings are shown in Supplemental Table 6 and Figure 10.

**Figure 11:**
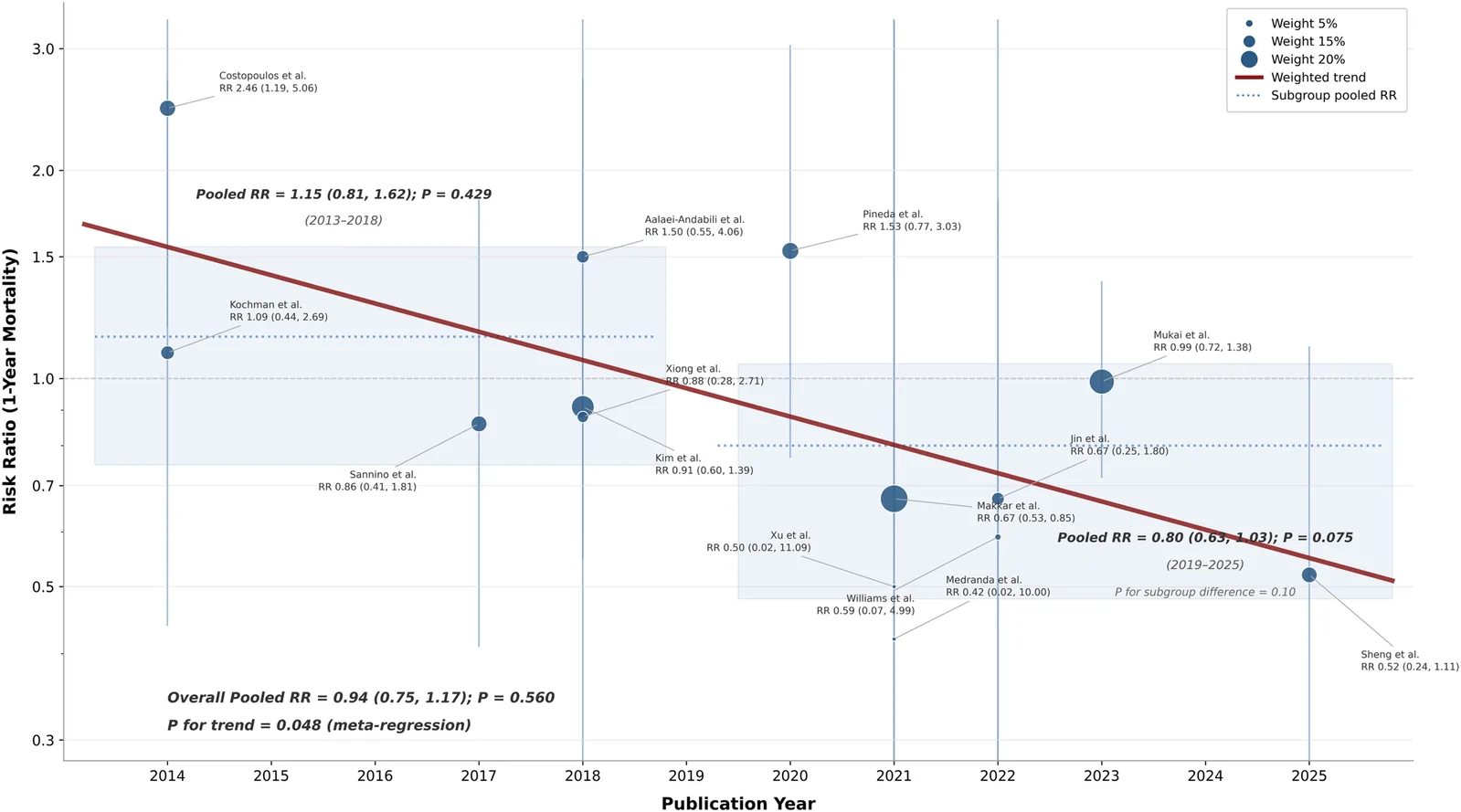
**Subgroup Analysis for 1-Year Mortality by Study Year** Subgroup analysis of 1-year mortality stratified by study year for transcatheter aortic valve replacement in bicuspid vs tricuspid aortic valve stenosis. Abbreviation as in Figure 2.

In the era-based subgroup analysis, studies published between 2013 and 2018 demonstrated a pooled RR for 1-year mortality of 1.15 (95% CI: 0.81-1.62; *P* = 0.429) comparing BAV to TAV, whereas studies published between 2019 and 2025 showed a pooled RR of 0.80 (95% CI: 0.63-1.03; *P* = 0.075) (Figure 11). Meta-regression evaluating the association between publication year and the relative risk of 1-year mortality demonstrated a significant temporal trend. Inverse-variance weighted meta-regression of log(RR) on publication year yielded a coefficient of −0.070 (95% CI: −0.139 to −0.001; *P* = 0.048) (Figure 11). This corresponds to an estimated 7% relative reduction in the RR per year (RR per year 0.93; 95% CI: 0.87-1.00), suggesting that outcomes for BAV relative to TAV have improved in more contemporary studies.

**Figure 12:**
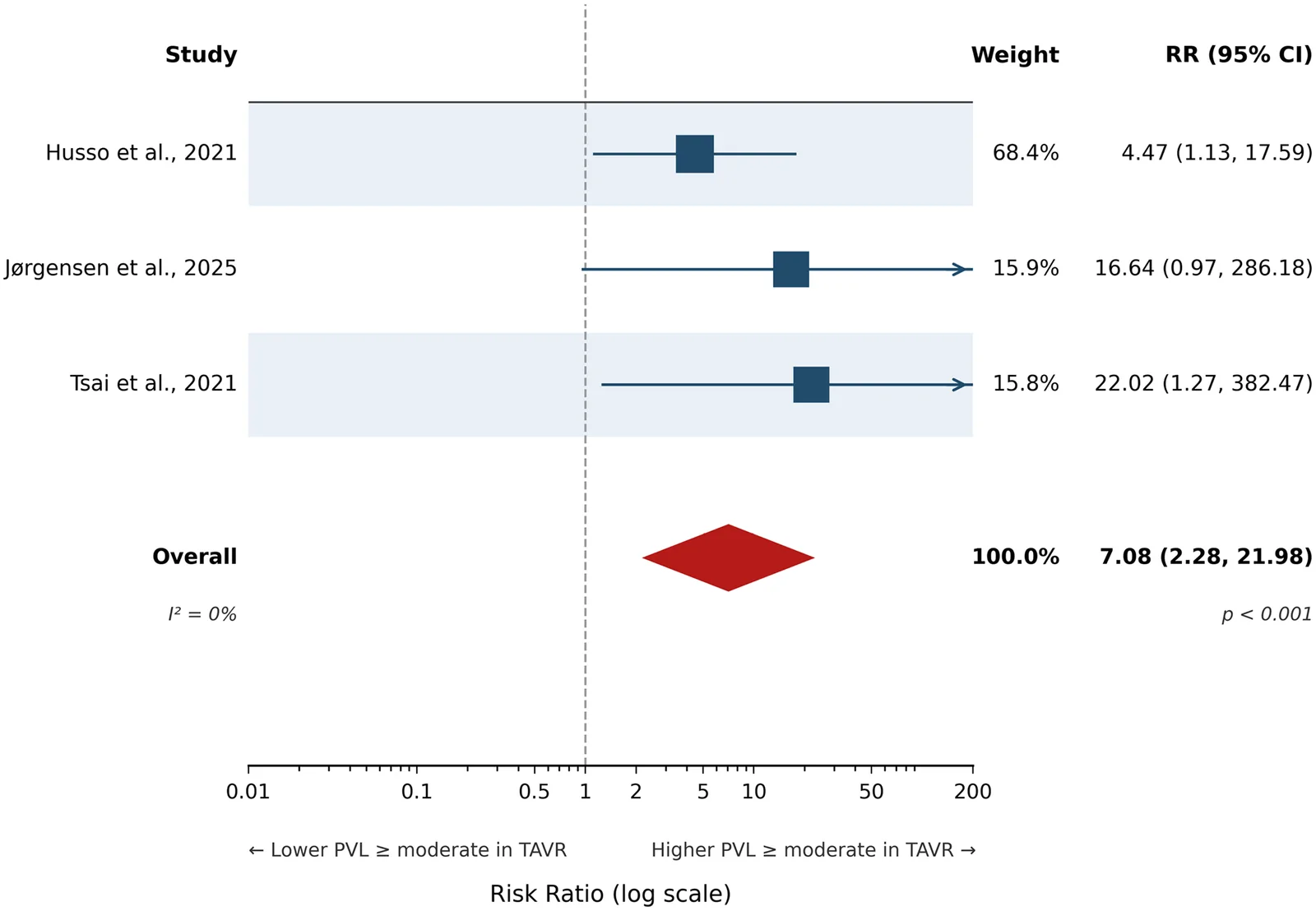
**Forest Plot for Moderate or Greater Paravalvular Leak (≥ Moderate) in Transcatheter Aortic Valve Replacement vs Surgical Aortic Valve Replacement for Bicuspid Aortic Valve Stenosis** Forest plot comparing moderate or greater paravalvular leak after transcatheter aortic valve replacement vs surgical aortic valve replacement in bicuspid aortic valve stenosis. TAVR = transcatheter aortic valve replacement; other abbreviations as in Figure 2.

**Figure 13:**
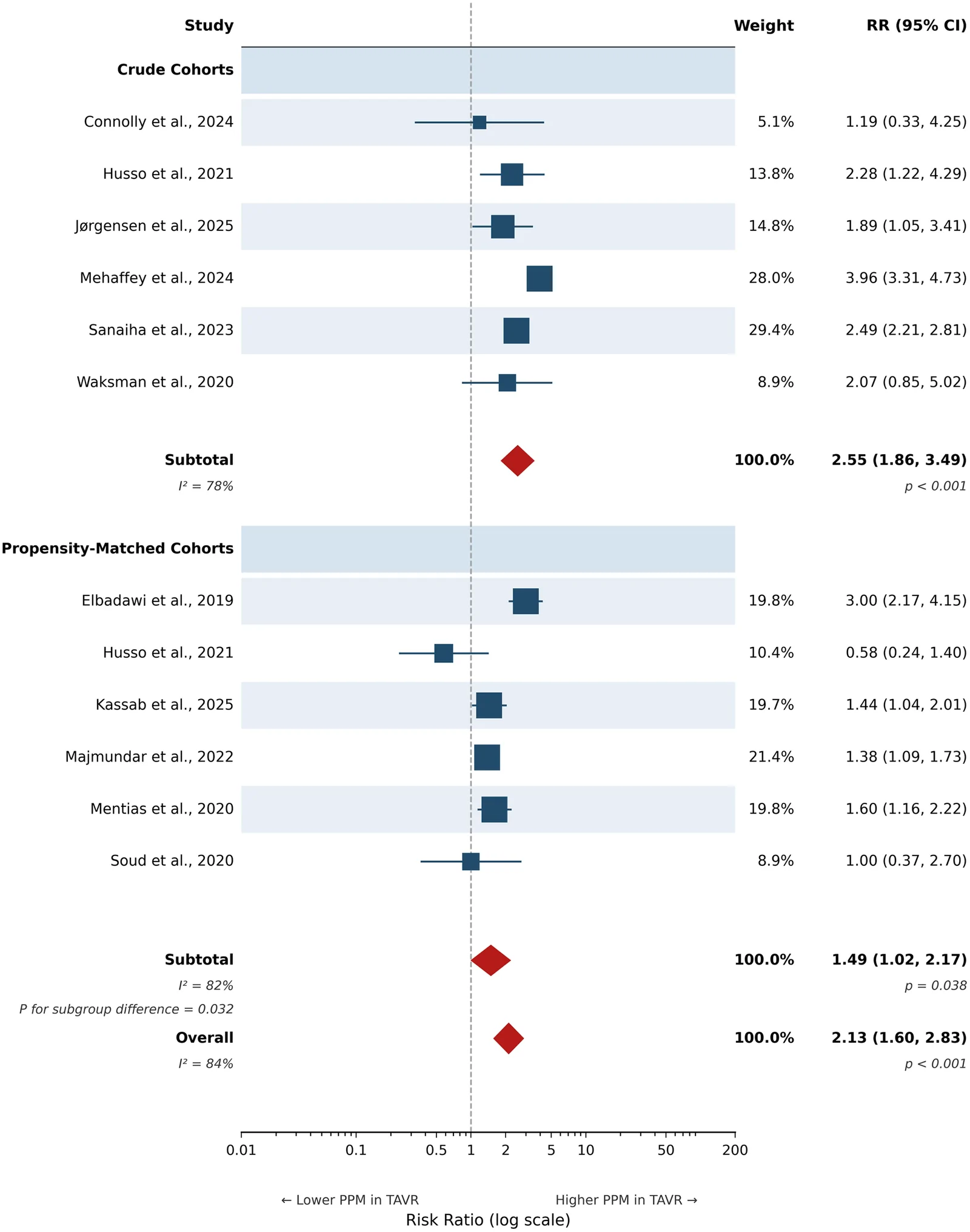
**Forest Plot for Permanent Pacemaker Implantation in Transcatheter Aortic Valve Replacement vs Surgical Aortic Valve Replacement for Bicuspid Aortic Valve Stenosis** Forest plot comparing rates of permanent pacemaker implantation after transcatheter aortic valve replacement vs surgical aortic valve replacement in bicuspid aortic valve stenosis. Abbreviations as in Figures 2, 3, and 12.

**Figure 14:**
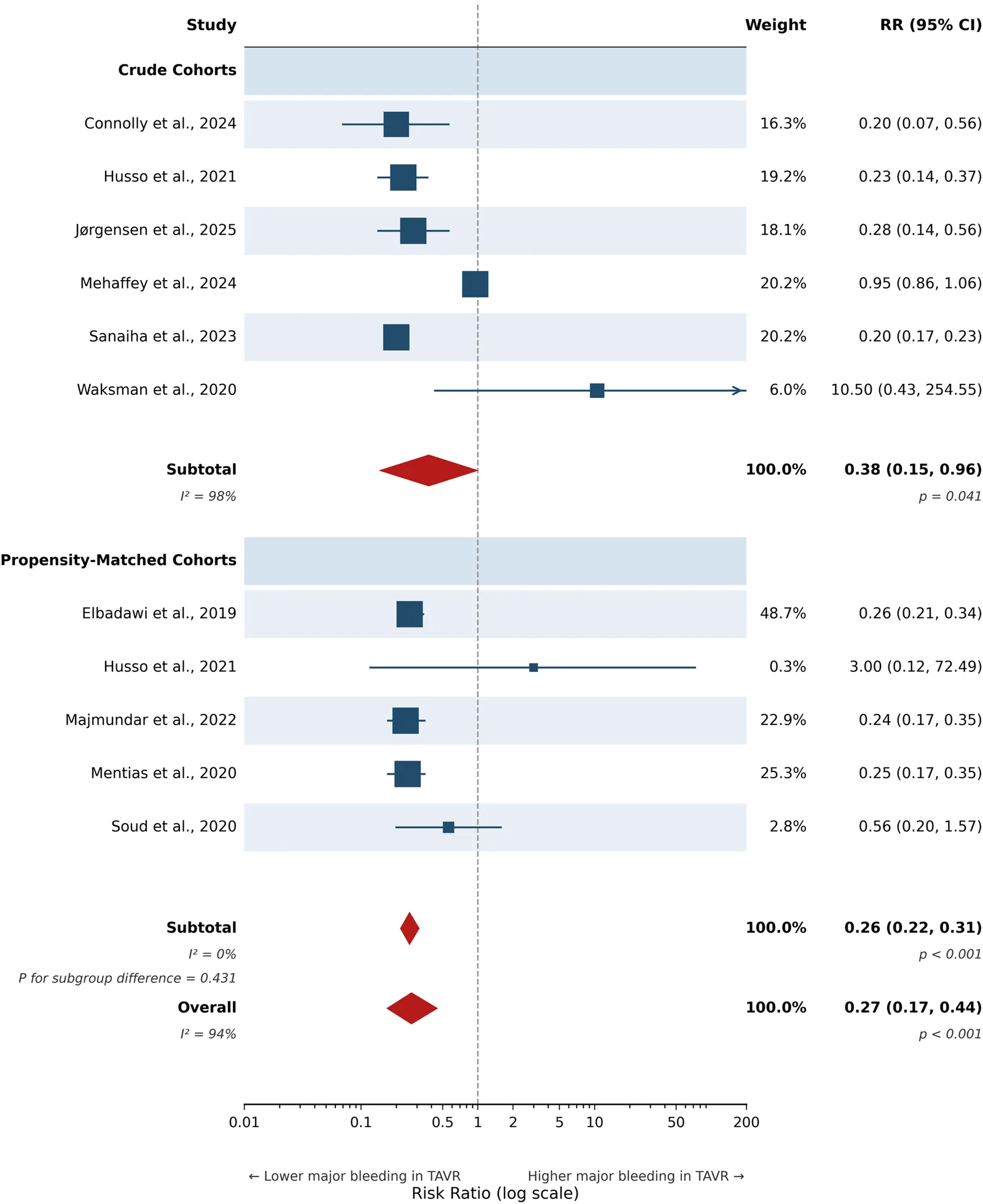
**Forest Plot for Major Bleeding in Transcatheter Aortic Valve Replacement vs Surgical Aortic Valve Replacement for Bicuspid Aortic Valve Stenosis** Forest plot comparing rates of major bleeding after transcatheter aortic valve replacement vs surgical aortic valve replacement in bicuspid aortic valve stenosis. Abbreviations as in Figures 2 and 12.

**Figure 15:**
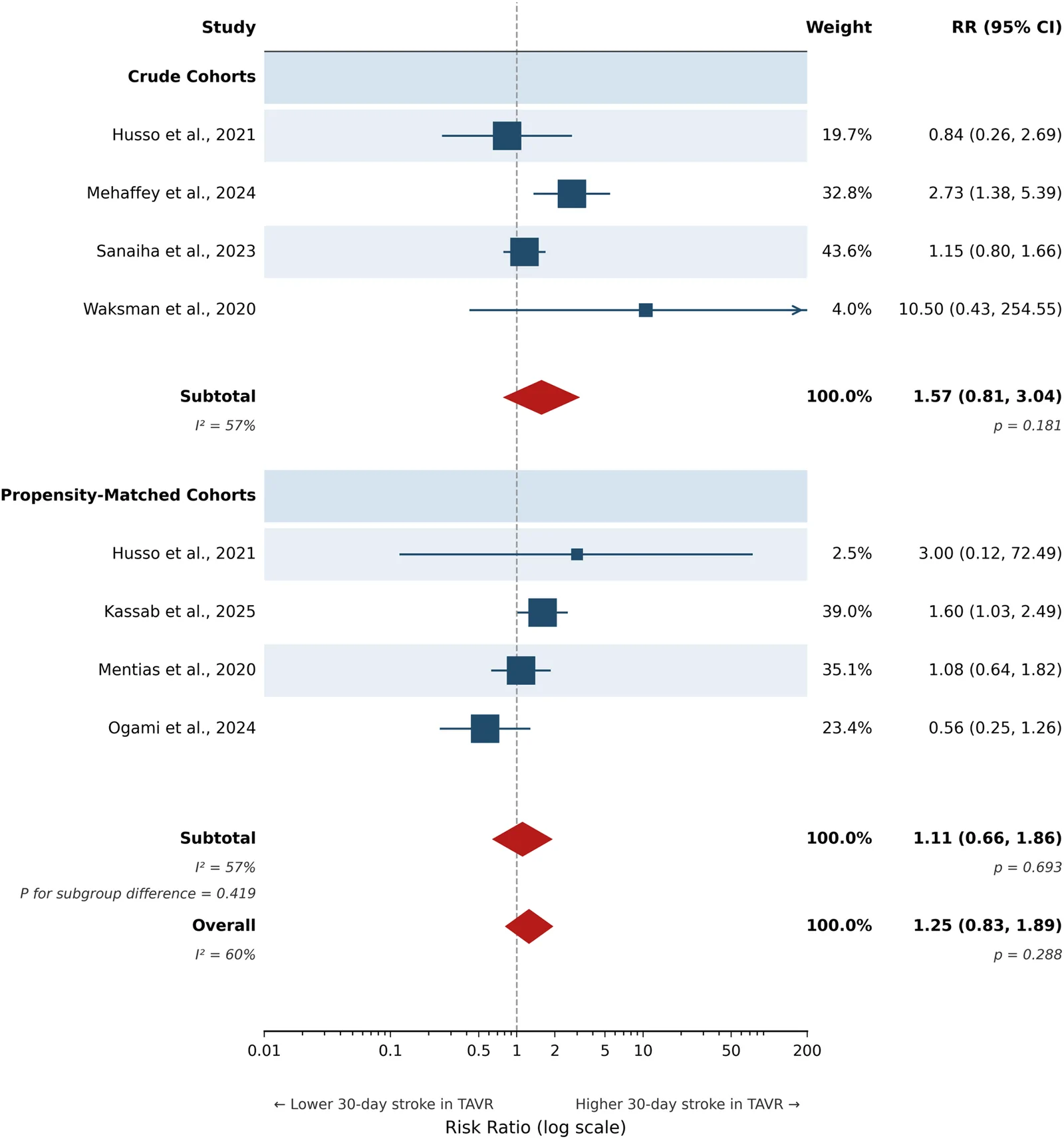
**Forest Plot for 30-Day Stroke in Transcatheter Aortic Valve Replacement vs Surgical Aortic Valve Replacement for Bicuspid Aortic Valve Stenosis** Forest plot comparing 30-day stroke after transcatheter aortic valve replacement vs surgical aortic valve replacement in bicuspid aortic valve stenosis. Abbreviations as in Figures 2 and 12.

**Figure 16:**
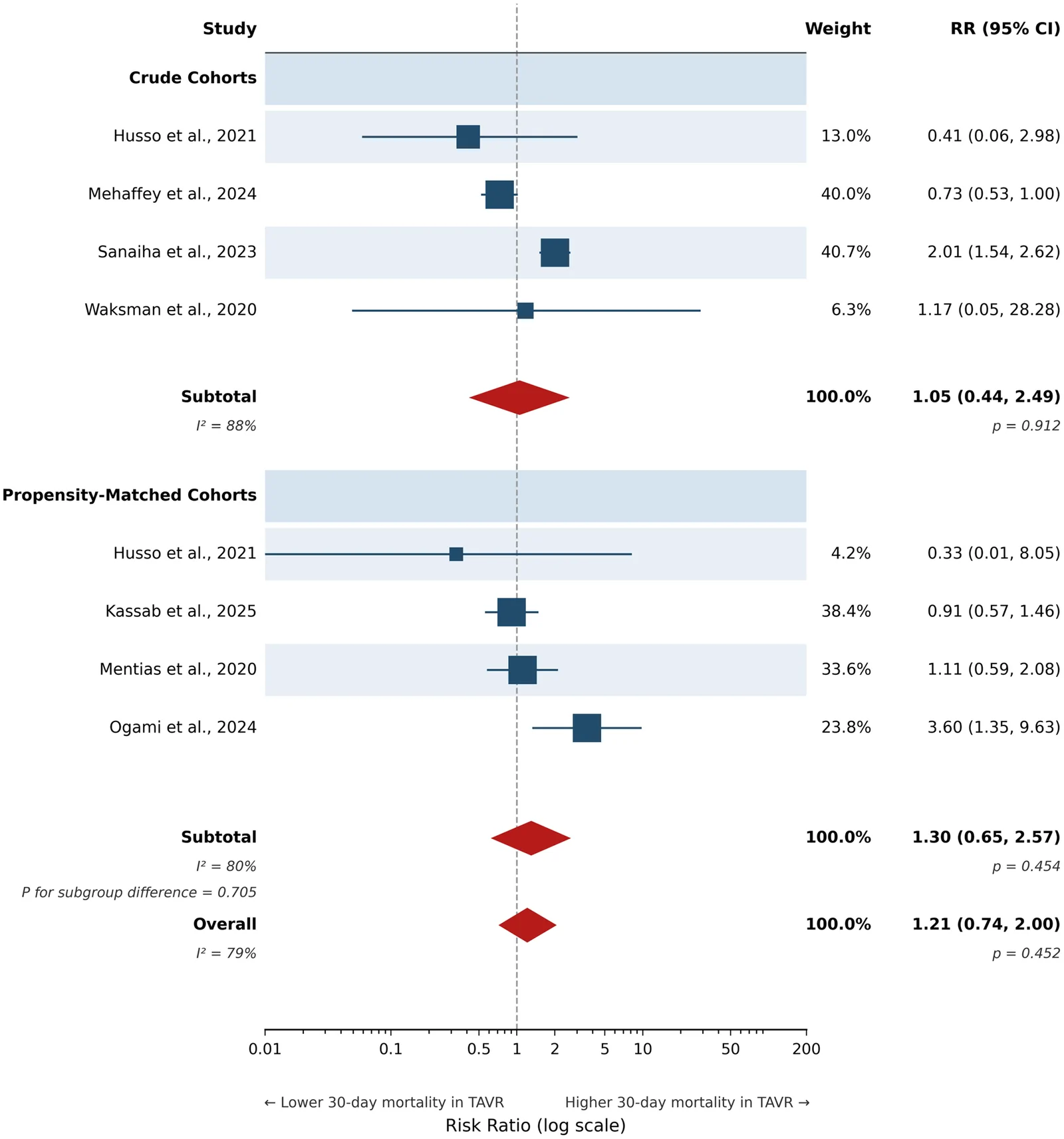
**Forest Plot for 30-Day Mortality in Transcatheter Aortic Valve Replacement vs Surgical Aortic Valve Replacement for Bicuspid Aortic Valve Stenosis** Forest plot comparing 30-day mortality after transcatheter aortic valve replacement vs surgical aortic valve replacement in bicuspid aortic valve stenosis. Abbreviations as in Figures 2 and 12.

Thirteen studies compared TAVR with SAVR in patients with BAV stenosis (Table 2).^49^, ^50^, ^51^, ^52^, ^53^, ^54^, ^55^, ^56^, ^57^, ^58^, ^59^, ^60^, ^61^ Baseline characteristics are summarized in Supplemental Table 7. Crude and propensity-matched subgroup results are presented separately in Supplemental Tables 8 and 9, respectively. Patients undergoing TAVR were significantly older than those undergoing SAVR (MD 3.48 years; 95% CI: 0.96-6.00; *P* = 0.007) (Supplemental Figure 5), and the proportion of male patients was lower in the TAVR group (RR: 0.77; 95% CI: 0.60-0.99; *P* = 0.041) (Supplemental Figure 6). In overall pooled analyses, TAVR was associated with substantially higher PVL (RR: 7.08; 95% CI: 2.28-21.98; *P* = 0.001) (Figure 12) and PPM (RR: 2.13; 95% CI: 1.60-2.83; *P* < 0.001) (Figure 13) than SAVR, whereas major bleeding was lower with TAVR (RR: 0.27; 95% CI: 0.17-0.44; *P* < 0.001) (Figure 14). Thirty-day stroke and 30-day mortality did not differ significantly between TAVR and SAVR in the overall pooled analyses (stroke: RR: 1.25; 95% CI: 0.83-1.89; *P* = 0.288 [Figure 15]; mortality: RR: 1.21; 95% CI: 0.74-2.00; *P* = 0.452 [Figure 16]).

**Table 2:** Studies for Comparative Outcomes of Transcatheter vs Surgical Aortic Valve Replacement in Bicuspid Aortic Valve Stenosis

First Author	Year	Number		Paravalvular Leak ≥ Moderate		Permanent Pacemaker Implantation		Aortic Root Injury^a^		Major Bleeding		30-Day Mortality		30-Day Stroke		1-Year Mortality		1-Year Stroke	
		TAVR	SAVR	TAVR	SAVR	TAVR	SAVR	TAVR	SAVR	TAVR	SAVR	TAVR	SAVR	TAVR	SAVR	TAVR	SAVR	TAVR	SAVR
Elbadawi	2019	975^b^	975^b^	NR	NR	135 (13.8%)	45 (4.6%)	NR	NR	70 (7.2%)	265 (27.2%)	NR	NR	NR	NR	NR	NR	NR	NR
Mentias	2020	699^b^	699^b^	NR	NR	85 (12.2%)	53 (7.6%)	NR	NR	37 (5.3%)	150 (21.5%)	20 (2.9%)	18 (2.7%)	28 (4.0%)	26 (3.7%)	63 (9.0%)	52 (7.4%)	28 (4.0%)	26 (3.7%)
Soud	2020	68^b^	68^b^	NR	NR	7 (10.3%)	7 (10.3%)	NR	NR	5 (7.4%)	9 (13.2%)	NR	NR	NR	NR	NR	NR	NR	NR
Waksman	2020	61	216	1 (1.6%)	NR	7 (11.5%)	12 (5.6%)	NR	NR	1 (1.6%)	0 (0%)	0 (0%)	1 (0.5%)	1 (1.6%)	0 (0%)	NR	NR	NR	NR
Husso	2021	75^b^	75^b^	0 (0%)	0 (0%)	8 (11.4%)	4 (5.5%)	0 (0%)	1 (1.3%)	2 (2.7%)	16 (21.3%)	1 (1.3%)	4 (5.3%)	3 (4.0%)	6 (8.0%)	NR	NR	NR	NR
Tsai	2021	48	82	6 (12.5%)	0 (0%)	NR	NR	NR	NR	0 (0%)	0 (0%)	NR	NR	NR	NR	NR	NR	NR	NR
Majmundar	2022	1,393^b^	1,393^b^	NR	NR	155/1,309 (11.8%)	113/1,314 (8.6%)	NR	NR	35 (2.5%)	145 (10.5%)	<10 (0.3%)	<10 (0.7%)	<10 (0.1%)	<10 (0.0%)	NR	NR	NR	NR
Sanaiha	2023	3,855	52,476	NR	NR	292 (7.6%)	1,597 (4.4%)	NR	NR	166 (4.3%)	11,440 (21.8%)	62 (1.6%)	420 (0.8%)	31 (0.8%)	367 (0.7%)	NR	NR	NR	NR
Ogami	2024	573^b^	573^b^	NR	NR	NR	NR	NR	NR	NR	NR	18 (3.2%)	5 (0.9%)	9 (1.6%)	16 (2.7%)	NR	NR	NR	NR
Connolly	2024	78	74	NR	NR	5 (6.4%)	4 (5.4%)	NR	NR	4 (5.1%)	19 (25.7%)	NR	NR	NR	NR	NR	NR	NR	NR
Kassab	2025	663^b^	663^b^	NR	NR	78 (11.8%)	54 (8.1%)	NR	NR	NR	NR	32 (4.8%)	35 (5.3%)	48 (7.3%)	30 (4.5%)	NR	NR	NR	NR
Jørgensen	2025	187	183	8 (4.7%)	0 (0%)	29 (15.1%)	15 (8%)	NR	NR	9 (4.8%)	32 (17.5%)	NR	NR	NR	NR	4 (2.1%)	2 (1.1%)	10 (5.4%)	3 (1.6%)
Mehaffey	2025	3,166	8,123	NR	NR	293 (12.4%)	190 (2.34%)	NR	NR	419 (13.2%)	1,127 (13.9%)	47 (1.48%)	166 (2.04%)	17 (0.54%)	16 (0.19%)	NR	NR	NR	NR
13 studies		11,841	65,600	14/310 (4.5%)	0/340 (0%)	1,094/11,136 (9.8%)	2094/64,866 (3.2%)	**–**	**–**	748/10,605 (7.1%)	13,203/64,364 (20.5%)	180/9,092 (2.0%)	649/62,825 (1.0%)	137/9,092 (1.5%)	461/62,825 (0.7%)	–	–	**–**	**–**
				7.08 (95% CI 2.28-21.98) *P* < 0.001		2.13 (95% CI 1.60-2.83) *P* < 0.001		***–***		0.27 (95% CI 0.17-0.44) *P* < 0.001		1.21 (95% CI 0.74-2.00) *P* = 0.452		1.25 (95% CI 0.83-1.89) *P* = 0.288		–		**–**	

SAVR = surgical aortic valve replacement; TAVR = transcatheter aortic valve replacement; other abbreviation as in Table 1.

a Aortic dissection and/or annulus rupture.

b Propensity-matched population.

**Central Illustration:**
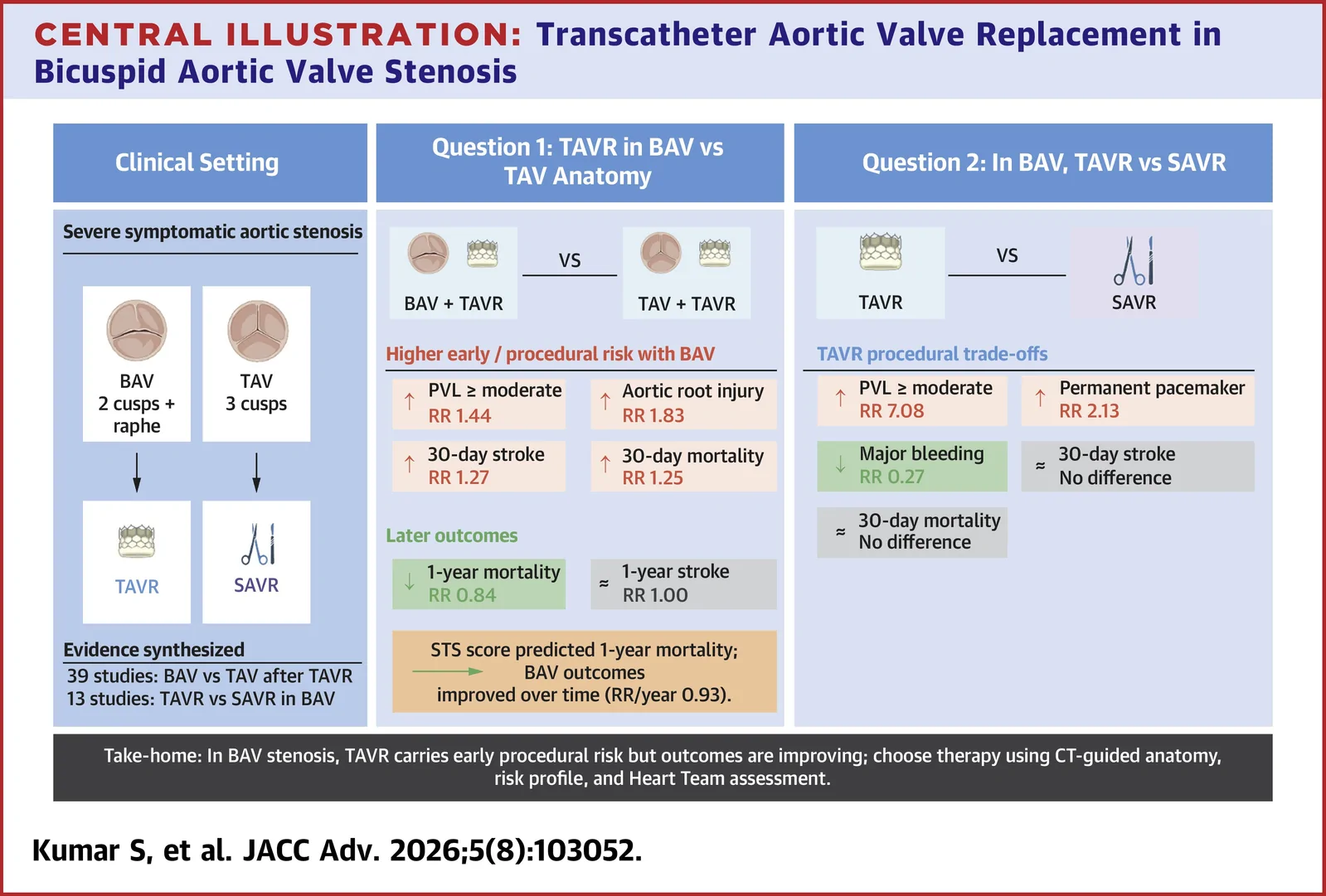
**Transcatheter Aortic Valve Replacement in Bicuspid Aortic Valve Stenosis** This systematic review and meta-analysis of 52 studies (295,615 patients) summarizes 2 clinical questions in severe bicuspid aortic valve stenosis. Compared with tricuspid anatomy, TAVR in bicuspid anatomy was associated with higher early procedural risk but lower 1-year mortality, with outcomes improving over time. Among patients with bicuspid stenosis, TAVR had higher paravalvular leak and pacemaker implantation but lower major bleeding than SAVR, with similar 30-day stroke and mortality, supporting individualized Heart Team decision-making. CT = computed tomography; SAVR = surgical aortic valve replacement; other abbreviations as in Figures 2, 10, and 12.

## Discussion

This updated systematic review and meta-analysis, which included 52 comparative studies with 295,615 patients, demonstrated that, compared with TAV, BAV anatomy was associated with a higher rate of periprocedural complications, including significant PVL, aortic root injury, 30-day stroke, and 30-day mortality after TAVR, but with lower 1-year mortality and a similar 1-year stroke rate. In addition, exploratory meta-regression analyses demonstrated that STS score—but not BAV anatomy—was independently associated with 1-year mortality and that 1-year outcomes of TAVR for BAV have improved over time. When treatment strategies within BAV were compared, TAVR was associated overall with higher PVL and PPM but lower major bleeding than SAVR, while early stroke and mortality were similar.

To our knowledge, this systematic review and meta-analysis represents the most up-to-date and largest synthesis of TAVR outcomes in BAV stenosis (Central Illustration). Unlike previous meta-analyses,^4^^,^^6^^,^^62^^,^^63^ our analysis uniquely incorporates both BAV-versus-TAV and TAVR-versus-SAVR comparisons, thereby addressing the 2 key clinical questions in BAV management: how TAVR performs in BAV compared with tricuspid anatomy and how TAVR compares with SAVR in patients with BAV. Saeed Al-Asad et al^4^ analyzed 30 studies including 193,274 TAVR patients, of whom 14,353 had BAV, and reported lower 1-year mortality but higher 30-day stroke rates in BAV. However, their study did not evaluate SAVR or aortic root injury, limiting assessment of treatment-strategy trade-offs. Consistent with their findings, we also observed a similar trend in 1-year mortality. Importantly, our meta-regression demonstrated that STS score, rather than BAV anatomy itself, was associated with 1-year mortality, suggesting that baseline patient risk may be more important than bicuspid anatomy alone in determining outcomes. Similarly, Gupta et al^6^ evaluated 50 studies including 203,288 patients and showed higher procedural risks in BAV, including mortality, stroke, PPM implantation, and PVL. Our analysis further extends these findings by demonstrating temporal improvement in outcomes over time, likely reflecting advances in procedural techniques and newer-generation transcatheter valve devices.

The excess risk of PVL and aortic root injury in TAVR for BAV is mechanistically consistent with the intrinsic structural characteristics of BAV anatomy. BAV is characterized by asymmetric leaflet fusion, calcified raphes, eccentric annular geometry, and frequently heavy leaflet-edge calcium.^64^^,^^65^ Deployment within an elliptical annulus, often combined with supra-annular constraint or tapered root morphology, can result in nonuniform radial force distribution, frame underexpansion, and incomplete sealing, all of which predispose to residual paravalvular regurgitation.^66^^,^^67^ In heavily calcified or raphe-dominant BAV, aggressive balloon dilatation or implantation of larger devices may further increase mechanical stress on the annulus and aortic root, contributing to a higher risk of aortic root injury.^68^^,^^69^ These anatomical and procedural complexities may also explain the higher rates of 30-day mortality and stroke observed in BAV. A greater calcium burden, more frequent balloon manipulations, and repeated recapture maneuvers with self-expandable valves may increase the likelihood of embolic events during the procedure.^70^^,^^71^ Therefore, these findings highlight the importance of careful patient selection, meticulous preprocedural planning, and procedural optimization in patients with BAV undergoing TAVR.

Fortunately, the early excess morbidity and mortality observed in BAV did not translate into higher 1-year event rates; in fact, the BAV cohort demonstrated lower 1-year mortality than the TAV cohort. However, this finding should be interpreted cautiously, given the inherent limitations of meta-analyses in adequately controlling for confounding factors. In particular, the meta-regression analysis identified the STS score, a surrogate of overall comorbidity burden, as an independent predictor of 1-year mortality. This observation suggests that patients with BAV undergoing TAVR are likely to be younger and less comorbid than those with TAV, which may partly explain the more favorable long-term outcomes despite greater anatomical and procedural complexity.

Recent interest in TAVR for BAV has increased following The Nordic Aortic Valve Intervention (NOTION-2) trial, in which the bicuspid subgroup showed numerically higher event rates with TAVR than with SAVR, although the differences were not statistically significant.^60^ However, in the present analysis, integrating the currently available evidence, no clear differences were observed between TAVR and SAVR in hard clinical endpoints among patients with BAV. Instead, the comparison demonstrated a trade-off in procedural complications: TAVR was associated with higher rates of PPM implantation and PVL, whereas SAVR was associated with a greater risk of major bleeding. These findings suggest that treatment selection for BAV should be individualized based on anatomical complexity, surgical and bleeding risks, and the long-term implications of pacemaker dependence. At the same time, the absence of adequately powered randomized trials should be acknowledged. Ongoing randomized, controlled studies, including the NAVIGATE Bicuspid (TraNscatheter Aortic Valve Implantation vs surGical AorTic valvE replacement in patients with Bicuspid aortic valve stenosis; NCT06440498) and BELIEVERS (Bicuspid aortic valve replacement: EvaLuatIon of transcathetEr VERsus Surgery; NCT07413965) trials, are expected to provide more definitive evidence on the optimal treatment strategy for patients with BAV stenosis.^72^

The temporal improvement in 1-year outcomes—an estimated 7% relative reduction in the mortality RR per year of publication—deserves emphasis. This trend likely reflects several converging advances: newer-generation devices with improved sealing skirts and repositioning capability; BAV-specific procedural strategies such as supra-annular sizing and refined balloon dilatation; routine multidetector computed tomography (MDCT)-guided patient selection and planning.^73^ Together, these developments suggest that contemporary outcomes of TAVR in BAV more closely approximate those in TAV.

### Study limitations

Several limitations should be considered when interpreting these findings. First, the majority of included studies were observational registries or retrospective cohort studies, and only a subset included propensity-matched analyses. Residual confounding and selection bias, therefore, cannot be excluded, particularly in comparisons between TAVR and SAVR, where treatment allocation was nonrandomized. Second, outcome definitions were not fully standardized across studies, and several outcomes were unavailable in all data sets. Third, although the overall pooled analyses were prespecified as the primary analysis to maximize the available evidence base, crude and matched analyses should be interpreted both separately and together, as they address complementary questions about overall contemporary practice and adjusted comparative risk. Fourth, patient-level imaging data were not available, preventing evaluation of anatomical predictors such as calcium burden, raphe morphology, membranous septum length, or supra-annular geometry. Fifth, the meta-regression analyses were exploratory, based on a limited number of studies for each endpoint, and subject to ecological bias because they used study-level rather than patient-level covariates. Sixth, because TAVR technology and procedural planning continue to evolve rapidly, these pooled estimates may partly reflect earlier-generation devices and historical practice patterns; moreover, relative risks should be interpreted alongside group denominators, event counts, and absolute event rates to better appreciate their clinical significance. Seventh, death may act as a competing event for later nonfatal outcomes, particularly 1-year stroke. Because individual patient-level time-to-event data and competing-risk adjusted estimates were not available, pooled estimates for nonfatal follow-up outcomes should be interpreted as endpoint-specific event risks rather than competing-risk adjusted effects. Likewise, for follow-up outcomes, particularly 1-year mortality and stroke, potential censoring and loss to follow-up could not be uniformly accounted for because individual patient-level time-to-event data and study-specific censoring information were unavailable. Finally, although we excluded overlapping data sets when identifiable, partial population overlap in large national registries cannot be entirely excluded.

## Conclusions

BAV was associated with higher periprocedural risks after TAVR compared with TAV, but a lower 1-year mortality rate. Compared with SAVR, TAVR showed characteristic procedural trade-offs without differences in mortality. These findings highlight the importance of careful preprocedural planning and procedural optimization in BAV anatomy and underscore the need for dedicated randomized trials.

Perspectives **COMPETENCY IN MEDICAL KNOWLEDGE:**

TAVR is an established treatment for severe symptomatic AS across surgical-risk groups, but evidence in BAV stenosis has remained less certain because patients with BAV were largely excluded from pivotal randomized TAVR trials.

**TRANSLATIONAL OUTLOOK:**

In this contemporary systematic review and meta-analysis of 52 comparative studies, TAVR in BAV was associated with higher periprocedural risks than TAVR in tricuspid anatomy, including significant PVL, aortic root injury, 30-day stroke, and 30-day mortality, but with lower 1-year mortality and similar 1-year stroke. Study-level meta-regression suggested that baseline surgical risk, as reflected by the STS score, rather than BAV anatomy itself, was associated with 1-year mortality and that outcomes in BAV appeared to improve over time. Compared with SAVR in patients with BAV, TAVR showed higher PVL and PPM, lower major bleeding, and no significant difference in early stroke or mortality. Future studies should focus on dedicated, adequately powered randomized trials comparing TAVR and SAVR in patients with BAV stenosis, particularly in younger and lower-risk populations.

## Funding support and author disclosures

The authors have reported that they have no relationships relevant to the contents of this paper to disclose.
