# Supplementary material for: Transcatheter Aortic Valve Replacement for Severe Bicuspid Aortic Valve Stenosis: A Systematic Review and Meta-Analysis
Source: JACC Adv. 2026 Aug 26;5(8):103052. doi: 10.1016/j.jacadv.2026.103052 (PMC13531625; doi:10.1016/j.jacadv.2026.103052)
Supplement: Supplementary file 1 [file mmc1.docx]

**SUPPLEMENTAL APPENDIX**

**Supplemental Table 1. Search strategy used in each database**

| Database | Search Strategy |
| --- | --- |
| MEDLINE | (("Bicuspid Aortic Valve"[MeSH] OR "bicuspid aortic valve"[All Fields] OR BAV[All Fields]) AND ("Aortic Valve Stenosis"[MeSH] OR "aortic stenosis"[All Fields])) AND (("Transcatheter Aortic Valve Replacement"[MeSH] OR "Transcatheter Aortic Valve Implantation"[MeSH] OR "transcatheter aortic valve replacement"[All Fields] OR "transcatheter aortic valve implantation"[All Fields] OR TAVR[All Fields] OR TAVI[All Fields]) OR ("Aortic Valve Replacement"[MeSH] OR "surgical aortic valve replacement"[All Fields] OR SAVR[All Fields])) |
| Scopus | (TITLE-ABS-KEY ("bicuspid aortic valve" OR BAV) AND TITLE-ABS-KEY ("aortic stenosis")) AND (TITLE-ABS-KEY ("transcatheter aortic valve replacement" OR "transcatheter aortic valve implantation" OR TAVR OR TAVI OR "surgical aortic valve replacement" OR SAVR)) |
| Cochrane CENTRAL | ("bicuspid aortic valve" OR BAV) AND ("aortic stenosis") AND ("transcatheter aortic valve replacement" OR "transcatheter aortic valve implantation" OR TAVR OR TAVI OR "surgical aortic valve replacement" OR SAVR) in Title, Abstract, Keywords |

**Supplemental Table 2. Modified Newcastle-Ottawa Scale (NOS) risk-of-bias assessment for individual studies**

| **Study** | **Representativeness of Exposed Cohort** | **Selection of Non-Exposed Cohort** | **Comparability at Baseline** | **Outcome Assessment** | **Sufficient Follow-Up** | **Adequacy of Follow-Up** | **Total (/6)** |
| --- | --- | --- | --- | --- | --- | --- | --- |
| Hayashida K (2013) | **★** | **★** | **★** | **★** | **★** | **–** | **5** |
| Bauer T (2014) | **★** | **★** | **★** | **★** | **★** | **★** | **6** |
| Costopoulos C (2014) | **★** | **★** | **★** | **★** | **★** | **–** | **5** |
| Kochman J (2014) | **★** | **★** | **★** | **★** | **★** | **–** | **5** |
| Liu XB (2015) | **–** | **★** | **★** | **★** | **–** | **–** | **3** |
| Watanabe Y (2015) | **–** | **★** | **★** | **★** | **–** | **–** | **3** |
| Arai T (2017) | **★** | **★** | **★** | **★** | **★** | **★** | **6** |
| Sannino A (2017) | **★** | **★** | **★** | **★** | **★** | **★** | **6** |
| Yoon SH (2017) | **★** | **★** | **★** | **★** | **★** | **★** | **6** |
| Liao YB (2018) | **–** | **★** | **★** | **★** | **★** | **–** | **4** |
| Aalaei-Andabili SH (2018) | **–** | **★** | **★** | **★** | **–** | **–** | **3** |
| De Biase C (2018) | **★** | **★** | **★** | **★** | **★** | **★** | **6** |
| Kim WK (2018) | **★** | **★** | **★** | **★** | **★** | **★** | **6** |
| Nagaraja V (2018) | **★** | **★** | **★** | **★** | **★** | **–** | **5** |
| Xiong TY (2018) | **–** | **★** | **★** | **★** | **–** | **–** | **3** |
| Mangieri A (2018) | **★** | **★** | **★** | **★** | **★** | **★** | **6** |
| Tchetche D (2019) | **★** | **★** | **★** | **★** | **★** | **★** | **6** |
| Elbadawi A (2019) | **★** | **★** | **★** | **★** | **–** | **–** | **4** |
| Pineda AM (2020) | **★** | **★** | **★** | **★** | **★** | **★** | **6** |
| Forrest JK (2020) | **★** | **★** | **★** | **★** | **★** | **★** | **6** |
| Halim SA (2020) | **★** | **★** | **★** | **★** | **–** | **–** | **4** |
| Mentias A (2020) | **★** | **★** | **★** | **★** | **–** | **–** | **4** |
| Soud M (2020) | **★** | **★** | **★** | **★** | **–** | **–** | **4** |
| Waksman R (2020) | **★** | **★** | **★** | **★** | **★** | **★** | **6** |
| Kim WK (2021) | **★** | **★** | **★** | **★** | **★** | **★** | **6** |
| Makkar RR (2021) | **★** | **★** | **★** | **★** | **★** | **★** | **6** |
| Medranda GA (2021) | **–** | **★** | **★** | **★** | **–** | **–** | **3** |
| Xu Q (2021) | **–** | **★** | **★** | **★** | **–** | **–** | **3** |
| Jung JH (2021) | **–** | **★** | **★** | **★** | **–** | **–** | **3** |
| Husso A (2021) | **★** | **★** | **★** | **★** | **★** | **★** | **6** |
| Tsai HY (2021) | **★** | **★** | **★** | **★** | **★** | **–** | **5** |
| Deeb GM (2022) | **★** | **★** | **★** | **★** | **★** | **★** | **6** |
| Williams MR (2022) | **★** | **★** | **★** | **★** | **★** | **★** | **6** |
| Fan J (2022) | **–** | **★** | **★** | **★** | **★** | **–** | **4** |
| Jin Q (2022) | **–** | **★** | **★** | **★** | **★** | **–** | **4** |
| Majmundar M (2022) | **★** | **★** | **★** | **★** | **–** | **–** | **4** |
| Hong N (2023) | **★** | **★** | **★** | **★** | **★** | **★** | **6** |
| Sanaiha Y (2023) | **★** | **★** | **★** | **★** | **–** | **–** | **4** |
| Zheng HJ (2023) | **★** | **★** | **★** | **★** | **★** | **★** | **6** |
| Ogami T (2024) | **★** | **★** | **★** | **★** | **★** | **–** | **5** |
| Connolly JE (2024) | **★** | **★** | **★** | **★** | **★** | **–** | **5** |
| Mehaffey JH (2024) | **★** | **★** | **★** | **★** | **★** | **★** | **6** |
| Liu K (2024) | **–** | **★** | **★** | **★** | **–** | **–** | **3** |
| Li HD (2024) | **–** | **★** | **★** | **★** | **★** | **–** | **4** |
| Kassab J (2025) | **–** | **★** | **★** | **★** | **–** | **–** | **3** |
| Jørgensen TH (2025) | **★** | **★** | **★** | **★** | **★** | **★** | **6** |
| Mehaffey JH (2025) | **★** | **★** | **★** | **★** | **★** | **★** | **6** |
| Yamanaka F (2025) | **★** | **★** | **★** | **★** | **★** | **★** | **6** |
| Zhu T (2025) | **–** | **★** | **★** | **★** | **–** | **–** | **3** |
| Sheng W (2025) | **–** | **★** | **★** | **★** | **–** | **–** | **3** |
| Louca A (2025) | **★** | **★** | **★** | **★** | **★** | **–** | **5** |
| Sun Y (2025) | **–** | **★** | **★** | **★** | **–** | **–** | **3** |

**Supplemental Table 3. Baseline Characteristics in Studies Comparing Transcatheter Aortic Valve Replacement in Bicuspid Versus Tricuspid Aortic Valve Stenosis**

| **First Author** | **Year** | **Number** | | **Age** | | **Male** | |
| --- | --- | --- | --- | --- | --- | --- | --- |
|  |  | **BAV** | **TAV** | **BAV** | **TAV** | **BAV** | **TAV** |
| **Hayashida K^1^** | 2013 | 21 | 208 | 82.0±7.0 | 83.2±6.5 | 12 (57.1%) | 111 (53.4%) |
| **Bauer T^2^** | 2014 | 38 | 1,357 | 80.7±6.6 | 81.8±6.2 | 17 (44.7%) | 570 (42.0%) |
| **Costopoulos C^3^** | 2014 | 21 | 447 | 76.7±7.1 | 79.8±7.4 | 12 (57.1%) | 212 (47.4%) |
| **Kochman J^4^** | 2014 | 28 | 84 | 77.6±5.5 | 79.1±6.8 | 13 (46.4%) | 40 (47.6%) |
| **Liu XB^5^** | 2015 | 15 | 25 | 75.4±5.7 | 75.8±5.5 | 9 (60.0%) | 17 (68.0%) |
| **Watanabe Y^6^** | 2015 | 11 | 56 | 82.5±4.6 | 84.4±6.0 | 7 (63.6%) | 37 (66.1%) |
| **Arai T^7^** | 2017 | 10 | 143 | 81.3±5.1 | 82.6±6.2 | 7 (70.0%) | 61 (42.7%) |
| **Sannino A^8^** | 2017 | 88 | 735 | 80.2±8.4 | 81.8±7.9 | 53 (60.2%) | 389 (52.9%) |
| **Yoon SH^9^** | 2017 | 546⁋ | 546⁋ | 77.2±8.2 | 77.2±8.8 | 343 (62.8%) | 331 (60.6%) |
| **Liao YB^10^** | 2018 | 87 | 70 | 73.4±6.4 | 74.3±7.0 | 50 (57.5%) | 45 (64.3%) |
| **Aalaei-Andabili SH^11^** | 2018 | 32 | 96 | 68.6±11.1 | 74.0±10.8 | 20 (66.7%) | 54 (56.3%) |
| **De Biase C^12^** | 2018 | 83⁋ | 166⁋ | 81.4±7.6 | 82.9±5.7 | 57 (68.7%) | 108 (65.1%) |
| **Kim WK^13^** | 2018 | 144 | 288 | *NR* | *NR* | *NR* | *NR* |
| **Nagaraja V^14^** | 2018 | 359⁋ | 359⁋ | 68.2±12.7 | 68.0±13.4 | 125 (34.8%) | 135 (37.6%) |
| **Xiong TY^15^** | 2018 | 67 | 49 | 74.0 (IQR 68.0–77.0) | 75.0 (IQR 68.0–79.0) | 40 (59.7%) | 28 (57.1%) |
| **Mangieri A^16^** | 2018 | 54 | 658 | 80±5.3 | 82.1±4.3 | 21 (38.9%) | 420 (63.9%) |
| **Tchetche D^17^** | 2019 | 101 | 88 | 78.2±10.1 | 83.1±5.7 | 66 (65.3%) | 41 (46.6%) |
| **Pineda AM^18^** | 2020 | 50 | 517 | 70 (IQR 64–74) | 81 (IQR 75–86) | 32 (64%) | 289 (55.9%) |
| **Forrest JK^19^** | 2020 | 929⁋ | 929⁋ | 73.0±10.3 | 72.6±10.8 | 512 (55.1%) | 508 (54.7%) |
| **Halim SA^20^** | 2020 | 5,412 | 165,547 | 74.0 (IQR 65.0–81.0) | 82.0 (IQR 76.0–87.0) | 3200 (59.1%) | 87474 (52.8%) |
| **Kim WK^21^** | 2021 | 242⁋ | 726⁋ | 80.0 (IQR 75.2–83.5) | 80.4 (IQR 76.7–83.5) | 152 (62.8%) | 440 (60.6%) |
| **Makkar RR^22^** | 2021 | 3,168⁋ | 3,168⁋ | 68.8±8.7 | 68.7±9.0 | 2190 (69.2%) | 2230 (70.4%) |
| **Medranda GA^23^** | 2021 | 47 | 60 | 68.4±7.8 | 76.0±5.7 | 21 (44.7%) | 37 (61.77%) |
| **Xu Q^24^** | 2021 | 9 | 14 | 71.2±4.8 | 76.9±5.7 | 3 (33.3%) | 9 (64.3%) |
| **Jung JH^25^** | 2021 | 19 | 48 | 73.2±7.2 | 77.8±5.8 | 12 (63.2%) | 23 (47.9%) |
| **Deeb GM^26^** | 2022 | 145⁋ | 145⁋ | 70.5±5.5 | 70.5±5.5 | 77 (53.1%) | 74 (51.0%) |
| **Williams MR^27(p3)^** | 2022 | 148⁋ | 148⁋ | 71.0 (IQR 68.0–75.0) | 72.0 (IQR 68.0–75.0) | 86 (58.1%) | 89 (60.1%) |
| **Fan J^28^** | 2022 | 223 | 172 | 72.0 (IQR 68.0–78.0) | 76.0 (IQR 70.0–82.0) | 137 (61.4%) | 85 (49.4%) |
| **Jin Q^29^** | 2022 | 195 | 149 | 74.8±7.3 | 77.1±9.3 | 96 (49.2%) | 77 (51.7%) |
| **Hong N^30^** | 2023 | 229 | 160 | 72.9±6.9 | 75.3±6.7 | 149 (65.1%) | 107 (66.9%) |
| **Mukai T**^31^ | 2023 | 423 | 16,802 | NR | NR | 225 (53.2%) | 5085 (30.3) |
| **Zheng HJ**^32^ | 2023 | 185 | 482 | 67 (IQR 65–76) | 78 (IQR 74–85) | 128 (69.2%) | 280 (58.1%) |
| **Liu K**^33^ | 2024 | 29 | 36 | 72.1±7.6 | 73.8±7.0 | 19 (65.5%) | 21 (58.3%) |
| **Li HD**^34^ | 2024 | 170 | 145 | 72.31±6.66 | 73.92±6.41 | 81 (47.6%) | 83 (57.2%) |
| **Yamanaka F**^35^ | 2025 | 497⁋ | 497⁋ | 84 (IQR 80–87) | 84 (IQR 80–87) | 195 (39.2%) | 180 (36.2%) |
| **Zhu T**^36^ | 2025 | 508 | 646 | 70.3±9.1 | 72.1±8.0 | 287 (56.5%) | 408 (63.2%) |
| **Sheng W**^37^ | 2025 | 193 | 234 | 74 (IQR 70–79) | 78 (IQR 72–82) | 46 (23.8%) | 60 (25.6%) |
| **Louca A**^38^ | 2025 | 577⁋ | 2,885⁋ | 76.8±7.8 | 80.9±6.4 | *NR* | *NR* |
| **Sun Y**^39^ | 2025 | 870⁋ | 870⁋ | 75 (IQR 70–80) | 75 (IQR 70–80) | 598 (68.7%) | 578 (66.4%) |
|  | 39 studies | 15,973 | 202,201 | 72.4±10.9 | 80.9±8.5 | 9021/15107 (59.7%) | 100562/196437 (51.2%) |
|  |  |  | | **Mean Difference −2.77 (95% CI −3.70, −1.84); p<0.001** | | **1.14 (95% CI 0.97–1.33); p=0.102** | |

Abbreviations: BAV = bicuspid aortic valve; CI = confidence interval; IQR = interquartile range; NR = not reported; TAV = tricuspid aortic valve

* Aortic dissection and/or annulus rupture † Lethal bleeding ‡ ≥ Mild ⁋ Propensity-matched population

| **Supplemental Table 4. Studies for Comparative Outcomes of Transcatheter Aortic Valve Replacement in Bicuspid Versus Tricuspid Aortic Valve Stenosis (Crude)** | | | | | | | | | | | | | | | | | | | |
| --- | --- | --- | --- | --- | --- | --- | --- | --- | --- | --- | --- | --- | --- | --- | --- | --- | --- | --- | --- |
| **First Author** | **Year** | **Number** | | **Paravalvular Leak ≥ Moderate** | | **Permanent Pacemaker Implantation** | | **Aortic Root Injury*** | | **Major Bleeding** | | **30-Day Mortality** | | **30-Day Stroke** | | **1-Year Mortality** | | **1-Year Stroke** | |
|  |  | **BAV** | **TAV** | **BAV** | **TAV** | **BAV** | **TAV** | **BAV** | **TAV** | **BAV** | **TAV** | **BAV** | **TAV** | **BAV** | **TAV** | **BAV** | **TAV** | **BAV** | **TAV** |
| Hayashida K | 2013 | 21 | 208 | 0 (0%) | 2 (1.0%) | 3 (14.3%) | 15 (7.2%) | 0 (0%) | 3 (1.4%) | 1 (4.8%) | 9 (4.3%) | 1 (4.8%) | 17 (8.2%) | *NR* | *NR* | *NR* | *NR* | *NR* | *NR* |
| Bauer T | 2014 | 38 | 1,357 | 10 (25%) | 204 (15%) | 6 (17%) | 475 (35%) | 1 (2.6%) | 5 (0.4%) | *NR* | *NR* | 4 (11%) | 149 (11%) | 0 (0%) | 41 (3%) | *NR* | *NR* | *NR* | *NR* |
| Costopoulos C | 2014 | 21 | 447 | 0 (0%) | 11 (3%) | 3 (14%) | 67 (15%) | *NR* | *NR* | 4 (19%) | 90 (20%) | 3 (16%) | 16 (3.6%) | 0 (0%) | 5 (1.0%) | 6 (32%) | 52 (14%) | *NR* | *NR* |
| Kochman J | 2014 | 28 | 84 | 9 (32%)‡ | 19 (23%)‡ | 8 (28%) | 28 (33%) | 0 (0%) | 0 (0%) | 3 (11%)† | 12 (14%)† | 1 (4%) | 6 (7%) | 0 (0%) | 3 (4%) | 5/23 (18%) | 14/70 (17%) | *NR* | *NR* |
| Liu XB | 2015 | 15 | 25 | 0 (0%) | 1 (4.0%) | 2 (13.3%) | 3 (12.0%) | 0 (0%) | 0 (0%) | 1 (6.7%) | 5 (20.0%) | 1 (6.7%) | 2 (8.0%) | 1 (6.7%) | 1 (4.0%) | *NR* | *NR* | *NR* | *NR* |
| Watanabe Y | 2015 | 11 | 56 | 5 (45.5%)‡ | 15 (26.8%)‡ | *NR* | *NR* | 0 (0%) | 2 (11.1%) | *NR* | *NR* | *NR* | *NR* | *NR* | *NR* | *NR* | *NR* | *NR* | *NR* |
| Arai T | 2017 | 10 | 143 | 0 (0%) | 8 (6%)‡ | 0 (0%) | 12 (8%) | 0 (0%) | 1 (1%) | 0 (0%) | 1 (1%)† | 0 (0%) | 1 (1%) | 0 (0%) | 0 (0%) | *NR* | *NR* | *NR* | *NR* |
| Sannino A | 2017 | 88 | 735 | 4 (5.3%) | 32 (5.0%) | 20 (22.7%) | 133 (18.2%) | *NR* | *NR* | 3 (3.4%) | 15 (2.0%) | 3 (3.4%) | 23 (3.1%) | 2 (2.3%) | 27 (3.7%) | 7 (8.5%) | 68 (10.5%) | *NR* | *NR* |
| Liao YB | 2018 | 87 | 70 | 1 (1.1%) | 0 (0%) | 21 (24.1%) | 20 (28.6%) | 0 (0%) | 0 (0%) | 12 (13.8%) | 7 (10%) | 8 (9.2%) | 3 (4.3%) | 1 (1.1%) | 0 (0%) | *NR* | *NR* | *NR* | *NR* |
| Aalaei-Andabili SH | 2018 | 32 | 96 | 1 (3.1%) | 2 (2.1%) | 4 (12.5%) | 13 (13.5%) | *NR* | *NR* | *NR* | *NR* | 2 (6.3%) | 4 (4.2%) | 2 (6.3%) | 3 (3.1%) | 5 (15.6%) | 10 (10.4%) | *NR* | *NR* |
| Kim WK | 2018 | 144 | 288 | 16 (11.1%) | 8 (2.8%) | *NR* | *NR* | 6 (4.2%) | 2 (0.7%) | *NR* | *NR* | 7 (4.9%) | 10 (3.5%) | *NR* | *NR* | 26 (18.2%) | 57 (19.8%) | *NR* | *NR* |
| Nagaraja V | 2018 | 407 | 40,197 | *NR* | *NR* | 40 (9.88%) | 4142 (10.30%) | *NR* | *NR* | 69 (16.96%) | 9445 (23.50%) | 20 (4.89%) | 1677 (4.17%) | 10 (2.49%) | 1425 (3.55%) | *NR* | *NR* | *NR* | *NR* |
| Xiong TY | 2018 | 67 | 49 | 4 (6.3%) | 3 (6.3%) | 17 (25.4%) | 11 (22.4%) | *NR* | *NR* | *NR* | *NR* | 6 (9.0%) | 2 (4.1%) | *NR* | *NR* | 6 (9.0%) | 5 (10.2%) | 1 (1.5%) | 1 (2.0%) |
| Mangieri A | 2018 | 54 | 658 | 4 (7.4%) | 9 (3.1%) | 5 (9.2%) | 57 (8.6%) | 0 (0%) | 0 (0%) | 2 (3.7%) | 34 (5.1%) | 2 (3.7%) | 17 (2.8%) | 4 (7.4%) | 12 (1.8%) | *NR* | *NR* | *NR* | *NR* |
| Tchetche D | 2019 | 101 | 88 | 21 (20.8%)‡ | 11 (12.5%)‡ | 13 (13%) | 12 (14%) | *NR* | *NR* | 11 (11%) | 4 (4.5%) | 0 (0%) | 3 (3.4%) | 2 (2%) | 0 (0%) | *NR* | *NR* | *NR* | *NR* |
| Pineda AM | 2020 | 50 | 517 | 2 (4.0%) | 19 (3.7%) | 4 (8%) | 73 (14.1%) | 1 (2%) | 3 (0.6%) | 1 (2.0%) | 1 (0.2%) | 3 (6.0%) | 8 (1.5%) | *NR* | *NR* | 8 (16.0%) | 54 (10.4%) | *NR* | *NR* |
| Halim SA | 2020 | 5,412 | 165,547 | 215/4856 (4.4%) | 4753/149,442 (3.2%) | *NR* | *NR* | *NR* | *NR* | 303/5335 (5.7%) | 10,042/163,278 (6.2%) | *NR* | *NR* | *NR* | *NR* | *NR* | *NR* | *NR* | *NR* |
| Makkar RR | 2021 | 3,243 | 34,417 | *NR* | *NR* | 232 (7.8%) | 2385 (7.7%) | 12 (0.4%) | 69 (0.2%) | *NR* | *NR* | 30 (1.0%) | 292 (0.9%) | 43 (1.4%) | 438 (1.3%) | 72 (4.5%) | 1136 (6.5%) | 50 (2.0%) | 604 (2.5%) |
| Medranda GA | 2021 | 47 | 60 | 0 (0%) | 1 (1.7%) | 3 (6.4%) | 1 (1.7%) | *NR* | *NR* | *NR* | *NR* | 0 (0%) | 0 (0%) | 1 (2.1%) | 0 (0%) | 0 (0%) | 1/59 (1.7%) | 2/43 (4.7%) | 1/58 (1.7%) |
| Xu Q | 2021 | 9 | 14 | 0 (0%) | 0 (0%) | *NR* | *NR* | *NR* | *NR* | *NR* | *NR* | 0 (0%) | 0 (0%) | 0 (0%) | 0 (0%) | 0 (0%) | 1 (7.1%) | *NR* | *NR* |
| Jung JH | 2021 | 19 | 48 | *NR* | *NR* | 0 (0%) | 2 (4.2%) | 0 (0%) | 0 (0%) | 0 (0%) | 1 (2.1%) | *NR* | *NR* | *NR* | *NR* | *NR* | *NR* | *NR* | *NR* |
| Williams MR | 2022 | 169 | 496 | *NR* | *NR* | 11 (6.5%) | 32 (6.5%) | *NR* | *NR* | *NR* | *NR* | 0 (0.0%) | 2 (0.4%) | 2 (1.2%) | 3 (0.6%) | 1 (0.6%) | 5 (1.0%) | 3 (1.8%) | 6 (1.2%) |
| Fan J | 2022 | 223 | 172 | *NR* | *NR* | 20 (9.0%) | 14 (8.1%) | 6 (2.7%) | 4 (2.3%) | *NR* | *NR* | 7 (3.3%) | 1 (0.6%) | 7 (3.3%) | 5 (3.1%) | *NR* | *NR* | *NR* | *NR* |
| Jin Q | 2022 | 195 | 149 | 27 (13.8%) | 10 (6.7%) | 25 (12.8%) | 22 (14.8%) | 0 (0%) | 1 (0.7%) | 2 (1.0%) | 2 (1.3%) | 3 (1.5%) | 4 (2.7%) | 10 (5.1%) | 3 (2.0%) | 7 (3.6%) | 8 (5.37%) | 12 (6.2%) | 5 (3.4%) |
| Hong N | 2023 | 229 | 160 | 6 (2.6%) | 4 (2.5%) | 15 (6.6%) | 14 (8.8%) | *NR* | N/A | 6 (2.6%) | 2 (1.3%) | 2 (0.9%) | 1 (0.7%) | 7 (3.3%) | 0 (0%) | *NR* | *NR* | *NR* | *NR* |
| Mukai T | 2023 | 423 | 16,802 | 13 (3.2%) | 290 (1.7%) | 19 (4.5%) | 880 (5.2%) | 0 (0%) | 46 (0.3%) | 1 (0.2%) | 54 (0.3%) | 2 (0.5%) | 214 (1.3%) | 8 (1.9%) | 213 (1.3%) | 34 (7.9%) | 1361 (8.1%) | *NR* | *NR* |
| Zheng HJ | 2023 | 185 | 482 | 6 (6.7%) | 13 (6.5%) | 8 (8.9%) | 20 (10.0%) | *NR* | *NR* | *NR* | *NR* | *NR* | *NR* | *NR* | *NR* | *NR* | *NR* | *NR* | *NR* |
| Liu K | 2024 | 29 | 36 | 2 (6.9%) | 2 (5.6%) | 1 (3.4%) | 5 (13.9%) | 0 (0%) | 0 (0%) | 0 (0%) | 0 (0%) | *NR* | *NR* | *NR* | *NR* | *NR* | *NR* | *NR* | *NR* |
| Li HD | 2024 | 170 | 145 | 3 (1.8%) | 0 (0%) | 43 (25.7%) | 34 (24.8%) | *NR* | *NR* | 17 (10.0%) | 6 (4.2%) | *NR* | *NR* | *NR* | *NR* | *NR* | *NR* | *NR* | *NR* |
| Zhu T | 2025 | 508 | 646 | 43 (8.5%) | 86 (13.3%) | 67 (13.2%) | 67 (10.4%) | *NR* | *NR* | *NR* | *NR* | *NR* | *NR* | *NR* | *NR* | *NR* | *NR* | *NR* | *NR* |
| Sheng W | 2025 | 193 | 234 | 4 (2.1%) | 6 (2.6%) | 9 (4.7%) | 28 (12.0%) | 0 (0%) | 0 (0%) | 21 (10.9%) | 28 (12.0%) | *NR* | *NR* | *NR* | *NR* | 9 (4.7%) | 21 (9.0%) | 5 (2.6%) | 4 (1.7%) |
| Louca A | 2025 | 577 | 6,518 | 25 (4.3%) | 162 (2.5%) | 67 (12%) | 462 (7.1%) | *NR* | *NR* | *NR* | *NR* | 10 (1.7%) | 122 (1.9%) | 3 (0.5%) | 60 (0.9%) | *NR* | *NR* | *NR* | *NR* |
| **32 studies** |  | **12,805** | **270,944** | **421/8188 (5.1%)** | **5671/179,509 (3.2%)** | **666/7229 (9.2%)** | **9027/105039 (8.6%)** | **20/4560 (0.4%)** | **132/55092 (0.2%)** | **457/7475 (6.1%)** | **19759/224,024 (8.8%)** | **114/6278 (1.8%)** | **2580/103750 (2.5%)** | **103/5996 (1.7%)** | **2239/102688 (2.2%)** | **186/4704 (4.0%)** | **2793/54373 (5.1%)** | **73/3910 (1.7%)** | **621/35403 (2.2%)** |
|  | | **283,749** | | ***RR 1.46 (95% CI 1.19–1.79)***  ***p<0.001*** | | ***RR 1.02 (95% CI 0.90–1.15)***  ***p=0.751*** | | ***RR 2.08 (95% CI 1.27–3.42)***  ***p=0.004*** | | ***RR 0.94 (95% CI 0.79–1.12)***  ***p=0.487*** | | ***RR 1.16 (95% CI 0.89–1.51)***  ***p=0.271*** | | ***RR 1.16 (95% CI 0.90–1.50)***  ***p=0.255*** | | ***RR 0.93 (95% CI 0.75–1.17)***  ***p=0.522*** | | ***RR 0.97 (95% CI 0.75–1.26)***  ***p=0.818*** | |

**Abbreviations:** BAV = bicuspid aortic valve; CI = confidence interval; IQR = interquartile range; NR = not reported; RR = relative risk; TAV = tricuspid aortic valve

| **Supplemental Table 5. Studies for Comparative Outcomes of Transcatheter Aortic Valve Replacement in Bicuspid Versus Tricuspid Aortic Valve Stenosis (Propensity-Matched)** | | | | | | | | | | | | | | | | | | | | |
| --- | --- | --- | --- | --- | --- | --- | --- | --- | --- | --- | --- | --- | --- | --- | --- | --- | --- | --- | --- | --- |
| **First Author** | **Year** | **Number** | | **Paravalvular Leak ≥ Moderate** | | **Permanent Pacemaker Implantation** | | **Aortic Root Injury*** | | **Major Bleeding** | | **30-Day Mortality** | | **30-Day Stroke** | | **1-Year Mortality** | | **1-Year Stroke** | |  |
|  |  | **BAV** | **TAV** | **BAV** | **TAV** | **BAV** | **TAV** | **BAV** | **TAV** | **BAV** | **TAV** | **BAV** | **TAV** | **BAV** | **TAV** | **BAV** | **TAV** | **BAV** | **TAV** |  |
| Yoon SH | 2017 | 546⁋ | 546⁋ | 57 (10.4%) | 37 (6.8%) | 84 (15.4%) | 84 (15.4%) | 9 (1.6%) | 0 (0%) | 20 (3.7%) | 22 (4.0%) | 20 (3.7%) | 18 (3.3%) | 16 (2.9%) | 10 (1.8%) | 66 (12.1%) | 73 (13.4%) | *NR* | *NR* |  |
| De Biase C | 2018 | 83⁋ | 166⁋ | 3 (3%) | 4 (2%) | 12 (14%) | 17 (10%) | 1 (1.2%) | 0 (0%) | *NR* | *NR* | 4 (5%) | 5 (3%) | 0 (0%) | 1 (0.6%) | *NR* | *NR* | *NR* | *NR* |  |
| Nagaraja V | 2018 | 359⁋ | 359⁋ | *NR* | *NR* | 40 (11.1%) | 15 (4.2%) | *NR* | *NR* | 64 (17.8%) | 85 (23.7%) | 20 (5.6%) | 5 (1.4%) | 10 (2.8%) | 20 (5.6%) | *NR* | *NR* | *NR* | *NR* |  |
| Forrest JK | 2020 | 929⁋ | 929⁋ | 47 (5.6%) | 18 (2.1%) | 133 (14.3%) | 115 (12.4%) | *NR* | *NR* | 1 (0.1%) | 1 (0.1%) | 23 (2.6%) | 15 (1.7%) | 31 (3.4%) | 25 (2.7%) | 62 (10.4%) | 69 (12.4%) | 33 (3.9%) | 34 (4.4%) |  |
| Kim WK | 2021 | 242⁋ | 726⁋ | 5 (2.1%) | 17 (2.3%) | 38 (15.7%) | 91 (12.5%) | 3 (1.2%) | 5 (0.7%) | 17 (7.0%) | 51 (7.0%) | 4 (1.7%) | 19 (2.6%) | 9 (3.7%) | 21 (2.9%) | *NR* | *NR* | *NR* | *NR* |  |
| Makkar RR | 2021 | 3,168⁋ | 3,168⁋ | 38 (1.8%) | 24 (1.1%) | 189 (6.2%) | 156 (5.2%) | 12 (0.4%) | 9 (0.3%) | 0 (0%) | 0 (0%) | 28 (0.9%) | 24 (0.8%) | 42 (1.4%) | 37 (1.2%) | 70 (4.6%) | 101 (6.6%) | 49 (2.0%) | 49 (2.0%) |  |
| Deeb GM | 2022 | 145⁋ | 145⁋ | 2 (1.4%) | 3 (2.1%) | 21 (14.9%) | 25 (17.2%) | *NR* | *NR* | 2 (1.4%) | 4 (2.8%) | 1 (0.7%) | 0 (0%) | 6 (4.1%) | 3 (2.1%) | 1 (0.7%) | 3 (2.1%) | 7 (4.8%) | 4 (2.8%) |  |
| Williams MR | 2022 | 148⁋ | 148⁋ | *NR* | *NR* | 9 (6.1%) | 10 (6.8%) | *NR* | *NR* | *NR* | *NR* | 0 (0%) | 0 (0%) | 2 (1.4%) | 2 (1.4%) | 1 (0.7%) | 2 (1.4%) | 3 (2.0%) | 3 (2.0%) |  |
| Yamanaka F | 2025 | 497⁋ | 497⁋ | 11 (2.2%) | 18 (3.6%) | 38 (7.7%) | 44 (8.9%) | 0 (0%) | 2 (2.0%) | 15 (3.0%) | 36 (7.2%) | 5 (1.0%) | 5 (1.0%) | *NR* | *NR* | *NR* | *NR* | *NR* | *NR* |  |
| Louca A | 2025 | 577⁋ | 2,885⁋ | 25 (4.3%) | 78 (2.7%) | 67 (12%) | 188 (6.9%) | *NR* | *NR* | *NR* | *NR* | 10 (1.7%) | 58 (2.0%) | *NR* | *NR* | *NR* | *NR* | *NR* | *NR* |  |
| Sun Y | 2025 | 870⁋ | 870⁋ | 32/722 (4.4%) | 32/735 (4.4%) | 61 (7.0%) | 45 (5.2%) | 3 (0.3%) | 3 (0.3%) | 10 (1.1%) | 3 (0.3%) | *NR* | *NR* | *NR* | *NR* | 38 (4.3%) | 46 (5.3%) | 13 (1.5%) | 12 (1.4%) |  |
| **11 studies** |  | **7,564** | **10,439** | **220/6,909 (3.2%)** | **231/9,797 (2.4%)** | **692/7,564 (9.1%)** | **790/10,439 (7.6%)** | **28/5,406 (0.5%)** | **19/5,973 (0.3%)** | **129/3,588 (3.6%)** | **202/4,072 (5.0%)** | **115/6,704 (1.7%)** | **149/9,569 (1.6%)** | **116/5,620 (2.1%)** | **129/6,187 (2.1%)** | **238/5806**  **(4.1%)** | **294/5806**  **(5.1%)** | **105/5260 (2.0%)** | **102/5260**  **(1.9%)** |  |
|  | | **18,003** | | ***RR 1.36 (95% CI 1.03–1.80)***  ***p=0.030*** | | ***RR 1.24 (95% CI 1.05–1.46)***  ***p=0.011*** | | ***RR 1.55 (95% CI 0.74–3.25)***  ***p=0.244*** | | ***RR 0.80 (95% CI 0.56–1.14)***  ***p=0.216*** | | ***RR 1.24 (95% CI 0.92–1.69)***  ***p=0.158*** | | ***RR 1.07 (95% CI 0.83–1.39)***  ***p=0.602*** | | ***RR 0.81 (95% CI 0.69–0.96)***  ***p=0.023*** | | ***RR 1.03 (95% CI 0.78–1.35)***  ***p=0.841*** | |  |

**Abbreviations:** BAV = bicuspid aortic valve; CI = confidence interval; IQR = interquartile range; NR = not reported; RR = relative risk; TAV = tricuspid aortic valve
** Aortic dissection and/or annulus rupture † Lethal bleeding ‡ ≥ Mild ⁋ Propensity-matched population*
*1-Year Mortality and 1-Year Stroke columns — populate with study-specific data as available.*

| **Supplemental Table 6. Meta Regression Analysis Data** | | | | | | | | |
| --- | --- | --- | --- | --- | --- | --- | --- | --- |
| **First Author/Study** | **Year** | **Age, years** | **Male** | **STS (%)** | **BEV** | **SEV** |  |  |
| **Hayashida K**^1^ | 2013 | 82.0 ± 7.0 | 12 (57.1%) | *NR* | 10 (47.6%) | 11 (52.4%) |  |  |
| **Bauer T**^2^ | 2014 | 80.7 ± 6.6 | 17 (44.7%) | *NR* | 26 (68%) | 12 (32%) |  |  |
| **Costopoulos C**^3^ | 2014 | 76.7 ± 7.1 | 12 (57.1%) | 7.6 ± 4.2 | 13 (62%) | 8 (38%) |  |  |
| **Kochman J**^4^ | 2014 | 77.6 ± 5.5 | 13 (46.4%) | *NR* | 23 (82%) | 5 (18%) |  |  |
| **Liu XB**^5^ | 2015 | 75.4 ± 5.7 | 9 (60.0%) | 5.6 ± 4.1 | 10 (66.7%) | 5 (33.3%) |  |  |
| **Watanabe Y**^6^ | 2015 | 82.5 ± 4.6 | 7 (63.6%) | *NR* | 0 (0%) | 6/6 (100%) |  |  |
| **Arai T**^7^ | 2017 | 81.3 ± 5.1 | 7 (70.0%) | *NR* | 10 (100%) | 0 (0%) |  |  |
| **Sannino A**^8^ | 2017 | 80.2 ± 8.4 | 53 (60.2%) | 7.4 ± 3.9% | 46 (52.3%) | 42 (47.7%) |  |  |
| **Liao YB**^10^ | 2018 | 73.4 ± 6.4 | 50 (57.5%) | 7.9 ± 4.0 | 0 (0%) | 87 (100%) |  |  |
| **Aalaei-Andabili SH**^11^ | 2018 | 68.6 ± 11.1 | 20 (66.7%) | 6.01 ± 3.42 | 25 (78.1%) | 7 (21.9%) |  |  |
| **Kim WK**^13^ | 2018 | *NR* | *NR* | *NR* | 91 (63.2%) | 46 (31.9%) |  |  |
| **Nagaraja V**^14^ | 2018 | 68.2 ± 12.7 | 125 (34.8%) | *NR* | *NR* | *NR* |  |  |
| **Xiong TY**^15^ | 2018 | 74.0  (IQR 68.0-77.0) | 40 (59.7%) | 6.5 (IQR 4.4-9.3) | 0 (0%) | 63 (94.0%) |  |  |
| **Mangieri A**^16^ | 2018 | 80 ± 5.3 | 21 (38.9%) | 4.7 ± 2.7 | 0 (0%) | 54 (100%) |  |  |
| **Tchetche D**^17^ | 2019 | 78.2 ± 10.1 | 66 (65.3%) | 11.3 ± 8.5 | 66 (65.3%) | 27 (26.7%) |  |  |
| **Pineda AM**^18^ | 2020 | 70 (IQR 64-74) | 32 (64%) | 4.6 (IQR 3.0-7.7) | 19 (38.0%) | 31 (62.0%) |  |  |
| **Halim SA**^20^ | 2020 | 74.0 (IQR 65.0–81.0) | 3200 (59.1%) | 3.8 (IQR 2.3-6.1) | 3551 (65.6%) | 827 (15.3%) |  |  |
| **Makkar RR**^22^ | 2021 | 68.8±8.7 | 2190 (69.2%) | 1.7 ± 0.6 | 3168 (100%) | 0 (0%) |  |  |
| **Medranda GA**^23^ | 2021 | 68.4 ± 7.8 | 21 (44.7%) | 1.5 ± 0.6 | 47 (100%) | 0 (0%) |  |  |
| **Xu Q**^24^ | 2021 | 71.2 ± 4.8 | 3 (33.3%) | 5.5 ± 3.0 | 0 (0%) | 0 (0%) |  |  |
| **Jung JH**^25^ | 2021 | 73.2 ± 7.2 | 12 (63.2%) | 2.6 (IQR 1.8-7.4) | 3 (15.8%) | 14 (73.7%) |  |  |
| **Williams MR**^27^ | 2022 | 71.0 (IQR 68.0-75.0) | 86 (58.1%) | 1.4 (IQR 1.0-1.9) | *NR* | *NR* |  |  |
| **Fan J**^28^ | 2022 | 72.0 (IQR 68.0-78.0) | 137 (61.4%) | 2.57 (IQR 1.57-4.58) | 26 (11.7%) | 197 (88.3%) |  |  |
| **Jin Q**^29^ | 2022 | 74.8 ± 7.3 | 96 (49.2%) | 4.3 ± 1.7 | 0 (0%) | 195 (100%) |  |  |
| **Hong N**^30^ | 2023 | 72.9 ± 6.9 | 149 (65.1%) | 2.6 ± 0.9 | 0 (0%) | 229 (100%) |  |  |
| **Mukai T**^31^ | 2023 | *NR* | 225 (53.2%) | 5.4 (IQR 3.5-8.2) | 239 (56.5%) | 184 (43.5%) |  |  |
| **Zheng HJ**^32^ | 2023 | 74 (IQR 67-82) | 418 (62.7%) | 5.6 (IQR 3.7-9.4) | *NR* | *NR* |  |  |
| **Liu K**^33^ | 2024 | 72.1 ± 7.6 | 19 (65.5%) | *NR* | 0 (0%) | 29 (100%) |  |  |
| **Li HD**^34^ | 2024 | 72.31±6.66 | 81 (47.6%) | 6.21 (IQR 3.66-8.60) | 5 (2.9%) | 155 (91.2%) |  |  |
| **Zhu T**^36^ | 2025 | 71.4 ± 10.2 | 695 (60.2%) | *NR* | *NR* | *NR* |  |  |
| **Sheng W**^37^ | 2025 | 76 (71–81) | 106 (24.8%） | 4.69 (2.72–8.79) | 56 (13.1%) | 361 (84.5%) |  |  |
| **Louca A**^38^ | 2025 | 80.6 (6.6%) | 3,819 (54%) | *NR* | 3150 (44.4%) | 3945 (55.6%) |  |  |
| **Sun Y** | 2025 | 74 (IQR 67-82) | 418 (62.7%) | *NR* | *NR* | *NR* |  |  |

Abbreviations: BAV = bicuspid aortic valve ; BEV = balloon-expandable valve; IQR = interquartile range;

NR = not reported; SEV = self-expanding valve; STS = society of thoracic surgeons; TAV = tricuspid aortic valve

**Supplemental Table 7. Baseline Characteristics in Studies Comparing Outcomes of Transcatheter vs. Surgical Aortic Valve Replacement in Bicuspid Aortic Valve Stenosis**

| **First Author** | **Year** | **Number** | | **Age** | | **Male** | |
| --- | --- | --- | --- | --- | --- | --- | --- |
|  |  | **TAVR** | **SAVR** | **TAVR** | **SAVR** | **TAVR** | **SAVR** |
| **Elbadawi A**^40^ | 2019 | 975⁋ | 975⁋ | 65.70±16.53 | 65.20±11.52 | 675 (69.2%) | 620 (63.6%) |
| **Mentias A**^41^ | 2020 | 699⁋ | 699⁋ | 74.7±9.4 | 69.9±6.8 | *NR* | *NR* |
| **Soud M**^42^ | 2020 | 68⁋ | 68⁋ | 65.0±14.8 | 64.6±12.4 | 22 (32.4%) | 49 (72.1%) |
| **Waksman R**^43^ | 2020 | 61 | 216 | 68.6±7.4 | 63.4±8.2 | 26 (42.6%) | 142 (65.7%) |
| **Husso A**^44^ | 2021 | 75⁋ | 75⁋ | 75.8±8.4 | 75.7±6.3 | 42 (56.0%) | 41 (54.7%) |
| **Tsai HY**^45^ | 2021 | 48 | 82 | 65.7±7.8 | 51.5±3.3 | 28 (58.3%) | 57 (69.5%) |
| **Majmundar M**^46^ | 2022 | 1,393⁋ | 1,393⁋ | 68.3±10.1 | 68.1±8.6 | 867 (62.2%) | 858 (61.6%) |
| **Sanaiha Y**^47^ | 2023 | 3,855 | 52,476 | 69 (IQR 62–76) | 59 (IQR 51–66) | 2359 (61.2%) | 36313 (69.2%) |
| **Ogami T**^48^ | 2024 | 573⁋ | 573⁋ | 74.0 (IQR 69.0–81.0) | 74.0 (IQR 69.0–79.0) | 165 (28.8%) | 161 (28.1%) |
| **Connolly JE**^49^ | 2024 | 78 | 74 | 74.1±8.93 | 66.0±8.29 | 42 (53.8%) | 47 (63.5%) |
| **Kassab J**^50^ | 2025 | 663⁋ | 663⁋ | 64.8±11.6 | 64.5±9.9 | 445 (67.1%) | 463 (69.8%) |
| **Jørgensen TH**^51^ | 2025 | 187 | 183 | 71.1±3.1 | 71.0±3.2 | 119 (63.6%) | 113 (61.7%) |
| **Mehaffey JH**^52^ | 2025 | 3,166 | 8,123 | 71 (IQR 67–75) | 69 (66–72) | 1829 (57.8%) | 5455 (67.2%) |
|  | 13 studies | 11,841 | 65,600 | 70.3±10.7 | 63.7±9.9 | 6619/11142 (59.4%) | 44319/64901 (68.3%) |
|  |  |  | | **Mean Difference 3.48 (95% CI 0.96–6.00); p=0.007** | | **0.77 (95% CI 0.60–0.99); p=0.041** | |

Abbreviations: CI = confidence interval; IQR = interquartile range; NR = not reported; SAVR = surgical aortic valve replacement; TAVR = transcatheter aortic valve replacement

| **Supplemental Table 8. Studies for Comparative Outcomes of Transcatheter vs. Surgical Aortic Valve Replacement in Bicuspid Aortic Valve Stenosis (Crude)** | | | | | | | | | | | | | | | | | | | |
| --- | --- | --- | --- | --- | --- | --- | --- | --- | --- | --- | --- | --- | --- | --- | --- | --- | --- | --- | --- |
| **First Author** | **Year** | **Number** | | **Paravalvular Leak ≥ Moderate** | | **Permanent Pacemaker Implantation** | | **Aortic Root Injury*** | | **Major Bleeding** | | **30-Day Mortality** | | **30-Day Stroke** | | **1-Year Mortality** | | **1-Year Stroke** | |
|  |  | **TAVR** | **SAVR** | **TAVR** | **SAVR** | **TAVR** | **SAVR** | **TAVR** | **SAVR** | **TAVR** | **SAVR** | **TAVR** | **SAVR** | **TAVR** | **SAVR** | **TAVR** | **SAVR** | **TAVR** | **SAVR** |
| Waksman R | 2020 | 61 | 216 | 1 (1.6%) | *NR* | 7 (11.5%) | 12 (5.6%) | *NR* | *NR* | 1 (1.6%) | 0 (0%) | 0 (0%) | 1 (0.5%) | 1 (1.6%) | 0 (0%) | *NR* | *NR* | *NR* | *NR* |
| Husso A | 2021 | 103 | 920 | 3 (2.9%) | 6 (0.7%) | 11 (11.6%) | 43 (4.8%) | 1 (1.0%) | 6 (0.7%) | 14 (14.9%) | 549 (60.1%) | 1 (0.9%) | 22 (2.4%) | 3 (2.9%) | 32 (3.5%) | *NR* | *NR* | *NR* | *NR* |
| Tsai HY | 2021 | 48 | 82 | 6 (12.5%) | 0 (0%) | *NR* | *NR* | *NR* | *NR* | 0 (0%) | 0 (0%) | *NR* | *NR* | *NR* | *NR* | *NR* | *NR* | *NR* | *NR* |
| Sanaiha Y | 2023 | 3,855 | 52,476 | *NR* | *NR* | 292 (7.6%) | 1597 (4.4%) | *NR* | *NR* | 166 (4.3%) | 11440 (21.8%) | 62 (1.6%) | 420 (0.8%) | 31 (0.8%) | 367 (0.7%) | *NR* | *NR* | *NR* | *NR* |
| Connolly JE | 2024 | 78 | 74 | *NR* | *NR* | 5 (6.4%) | 4 (5.4%) | *NR* | *NR* | 4 (5.1%) | 19 (25.7%) | *NR* | *NR* | *NR* | *NR* | *NR* | *NR* | *NR* | *NR* |
| Jørgensen TH | 2025 | 187 | 183 | 8 (4.7%) | 0 (0%) | 29 (15.1%) | 15 (8%) | *NR* | *NR* | 9 (4.8%) | 32 (17.5%) | *NR* | *NR* | *NR* | *NR* | 4 (2.1%) | 2 (1.1%) | 10 (5.4%) | 3 (1.6%) |
| Mehaffey JH | 2025 | 3,166 | 8,123 | *NR* | *NR* | 293 (12.4%) | 190 (2.34%) | *NR* | *NR* | 419 (13.2%) | 1127 (13.9%) | 47 (1.48%) | 166 (2.04%) | 17 (0.54%) | 16 (0.19%) | *NR* | *NR* | *NR* | *NR* |
| **7 studies** |  | **7,498** | **62,074** | **17/338 (5.0%)** | **6/1,185 (0.5%)** | **637/7,450 (8.6%)** | **1,861/61,992 (3.0%)** | **–** | **–** | **613/7,498 (8.2%)** | **13,167/62,074 (21.2%)** | **110/7,185 (1.5%)** | **609/61,735 (1.0%)** | **52/7,185 (0.7%)** | **415/61,735 (0.7%)** |  |  |  |  |
|  | | **69,572** | | ***RR 7.08 (95% CI 2.28–21.98)***  ***p=0.001*** | | ***RR 2.55 (95% CI 1.86–3.49)***  ***p<0.001*** | | *–* | | ***RR 0.38 (95% CI 0.15–0.96)***  ***p=0.041*** | | ***RR 1.05 (95% CI 0.44–2.49)***  ***p=0.912*** | | ***RR 1.57 (95% CI 0.81–3.04)***  ***p=0.179*** | | *–* | | *–* | |

**Abbreviations:** CI = confidence interval; IQR = interquartile range; NR = not reported; RR = relative risk; SAVR = surgical aortic valve replacement; TAVR = transcatheter aortic valve replacement
** Aortic dissection and/or annulus rupture † Lethal bleeding ‡ ≥ Mild*

| **Supplemental Table 9. Studies for Comparative Outcomes of Transcatheter vs. Surgical Aortic Valve Replacement in Bicuspid Aortic Valve Stenosis (Propensity-Matched)** | | | | | | | | | | | | | | | | | | | | |
| --- | --- | --- | --- | --- | --- | --- | --- | --- | --- | --- | --- | --- | --- | --- | --- | --- | --- | --- | --- | --- |
| **First Author** | **Year** | **Number** | | **Paravalvular Leak ≥ Moderate** | | **Permanent Pacemaker Implantation** | | **Aortic Root Injury*** | | **Major Bleeding** | | **30-Day Mortality** | | **30-Day Stroke** | | **1-Year Mortality** | | | **1-Year Stroke** | |
|  |  | **TAVR** | **SAVR** | **TAVR** | **SAVR** | **TAVR** | **SAVR** | **TAVR** | **SAVR** | **TAVR** | **SAVR** | **TAVR** | **SAVR** | **TAVR** | **SAVR** | **TAVR** | **SAVR** | **TAVR** | | **SAVR** |
| Elbadawi A | 2019 | 975⁋ | 975⁋ | *NR* | *NR* | 135 (13.8%) | 45 (4.6%) | *NR* | *NR* | 70 (7.2%) | 265 (27.2%) | *NR* | *NR* | *NR* | *NR* | *NR* | *NR* | *NR* | | *NR* |
| Mentias A | 2020 | 699⁋ | 699⁋ | *NR* | *NR* | 85 (12.2%) | 53 (7.6%) | *NR* | *NR* | 37 (5.3%) | 150 (21.5%) | 20 (2.9%) | 18 (2.7%) | 28 (4.0%) | 26 (3.7%) | 63 (9.0%) | 52 (7.4%) | 28 (4.0%) | | 26 (3.7%) |
| Soud M | 2020 | 68⁋ | 68⁋ | *NR* | *NR* | 7 (10.3%) | 7 (10.3%) | *NR* | *NR* | 5 (7.4%) | 9 (13.2%) | *NR* | *NR* | *NR* | *NR* | *NR* | *NR* | *NR* | | *NR* |
| Husso A | 2021 | 75⁋ | 75⁋ | 0 (0%) | 0 (0%) | 8 (11.4%) | 4 (5.5%) | 0 (0%) | 1 (1.3%) | 2 (2.7%) | 16 (21.3%) | 1 (1.3%) | 4 (5.3%) | 3 (4.0%) | 6 (8.0%) | *NR* | *NR* | *NR* | | *NR* |
| Majmundar M | 2022 | 1,393⁋ | 1,393⁋ | *NR* | *NR* | 155/1309 (11.8%) | 113/1314 (8.6%) | *NR* | *NR* | 35 (2.5%) | 145 (10.5%) | <10 (0.3%) | <10 (0.7%) | <10 (0.1%) | <10 (0.0%) | *NR* | *NR* | *NR* | | *NR* |
| Ogami T | 2024 | 573⁋ | 573⁋ | *NR* | *NR* | *NR* | *NR* | *NR* | *NR* | *NR* | *NR* | 18 (3.2%) | 5 (0.9%) | 9 (1.6%) | 16 (2.7%) | *NR* | *NR* | *NR* | | *NR* |
| Kassab J | 2025 | 663⁋ | 663⁋ | *NR* | *NR* | 78 (11.8%) | 54 (8.1%) | *NR* | *NR* | *NR* | *NR* | 32 (4.8%) | 35 (5.3%) | 48 (7.3%) | 30 (4.5%) | *NR* | *NR* | *NR* | | *NR* |
| **7 studies** |  | **4,446** | **4,446** | **–** | **–** | **467/3,789 (12.3%)** | **284/3,794 (7.5%)** | **–** | **–** | **148/3,210 (4.6%)** | **569/3,210 (17.7%)** | **70/2,010 (3.5%)** | **59/2,010 (2.9%)** | **86/2,010 (4.3%)** | **72/2,010 (3.6%)** | ***NR*** | ***NR*** | ***NR*** | | ***NR*** |
|  | | **8,892** | | *–* | | ***RR 1.49 (95% CI 1.02–2.17)***  ***p=0.040*** | | *–* | | ***RR 0.26 (95% CI 0.22–0.31)***  ***p<0.001*** | | ***RR 1.30 (95% CI 0.65–2.57)***  ***p=0.455*** | | ***RR 1.11 (95% CI 0.66–1.86)***  ***p=0.699*** | | *–* | | *–* | | |

**Abbreviations:** CI = confidence interval; IQR = interquartile range; NR = not reported; RR = relative risk; SAVR = surgical aortic valve replacement; TAVR = transcatheter aortic valve replacement

** Aortic dissection and/or annulus rupture † Lethal bleeding ‡ ≥ Mild ⁋ Propensity-matched population*
*1-Year Mortality and 1-Year Stroke columns — populate with study-specific data as available.*

**SUPPLEMENTAL FIGURE LEGEND**

**Supplemental Figure 1. Publication Bias Assessment in Transcatheter Aortic Valve Replacement for Bicuspid Aortic Valve vs. Tricuspid Aortic Valve Aortic Stenosis**

Funnel plot assessing publication bias in studies comparing TAVR outcomes in bicuspid versus tricuspid aortic valve stenosis.

**Supplemental Figure 2. Publication Bias Assessment in Transcatheter vs. Surgical Aortic Valve Replacement for Bicuspid Aortic Valve Aortic Stenosis**

Funnel plot assessing publication bias in studies comparing TAVR versus SAVR in bicuspid aortic valve stenosis.

**Supplemental Figure 3. Forest Plot for Age in Transcatheter Aortic Valve Replacement for Bicuspid Aortic Valve vs. Tricuspid Aortic Valve Aortic Stenosis**

Forest plot comparing age between bicuspid and tricuspid aortic valve stenosis cohorts undergoing TAVR.

**Supplemental Figure 4. Forest Plot for Sex in Transcatheter Aortic Valve Replacement for Bicuspid Aortic Valve vs. Tricuspid Aortic Valve Aortic Stenosis**

Forest plot comparing sex distribution between bicuspid and tricuspid aortic valve stenosis cohorts undergoing TAVR.

**Supplemental Figure 5. Forest Plot for Age in Transcatheter vs. Surgical Aortic Valve Replacement for Bicuspid Aortic Valve Aortic Stenosis**

Forest plot comparing age between TAVR and SAVR cohorts in bicuspid aortic valve stenosis.

**Supplemental Figure 6. Forest Plot for Sex in Transcatheter vs. Surgical Aortic Valve Replacement for Bicuspid Aortic Valve Aortic Stenosis**

Forest plot comparing sex distribution between TAVR and SAVR cohorts in bicuspid aortic valve stenosis.

**Supplemental Figure 1. Publication Bias Assessment in Transcatheter Aortic Valve Replacement for Bicuspid Aortic Valve vs. Tricuspid Aortic Valve Aortic Stenosis**

**Supplemental Figure 2. Publication Bias Assessment in Transcatheter vs. Surgical Aortic Valve Replacement for Bicuspid Aortic Valve Aortic Stenosis**

**Supplemental Figure 3. Forest Plot for Age in Transcatheter Aortic Valve Replacement for Bicuspid Aortic Valve vs. Tricuspid Aortic Valve Aortic Stenosis**

**Supplemental Figure 4. Forest Plot for Sex in Transcatheter Aortic Valve Replacement for Bicuspid Aortic Valve vs. Tricuspid Aortic Valve Aortic Stenosis**

**Supplemental Figure 5. Forest Plot for Age in Transcatheter vs. Surgical Aortic Valve Replacement for Bicuspid Aortic Valve Aortic Stenosis**

**Supplemental Figure 6. Forest Plot for Sex in Transcatheter vs. Surgical Aortic Valve Replacement for Bicuspid Aortic Valve Aortic Stenosis**

**REFERENCES**

1.	Hayashida K, Bouvier E, Lefèvre T, et al. Transcatheter aortic valve implantation for patients with severe bicuspid aortic valve stenosis. *Circ Cardiovasc Interv*. 2013;6(3):284-291. doi:10.1161/CIRCINTERVENTIONS.112.000084

2.	Bauer T, Linke A, Sievert H, et al. Comparison of the Effectiveness of Transcatheter Aortic Valve Implantation in Patients With Stenotic Bicuspid Versus Tricuspid Aortic Valves (from the German TAVI Registry). *The American Journal of Cardiology*. 2014;113(3):518-521. doi:10.1016/j.amjcard.2013.10.023

3.	Costopoulos C, Latib A, Maisano F, et al. Comparison of Results of Transcatheter Aortic Valve Implantation in Patients With Severely Stenotic Bicuspid Versus Tricuspid or Nonbicuspid Valves. *The American Journal of Cardiology*. 2014;113(8):1390-1393. doi:10.1016/j.amjcard.2014.01.412

4.	Kochman J, Huczek Z, Ścisło P, et al. Comparison of One- and 12-Month Outcomes of Transcatheter Aortic Valve Replacement in Patients With Severely Stenotic Bicuspid Versus Tricuspid Aortic Valves (Results from a Multicenter Registry). *The American Journal of Cardiology*. 2014;114(5):757-762. doi:10.1016/j.amjcard.2014.05.063

5.	Liu X bao, Jiang J bo, Zhou Q jing, et al. Evaluation of the safety and efficacy of transcatheter aortic valve implantation in patients with a severe stenotic bicuspid aortic valve in a Chinese population. *Journal of Zhejiang University Science B*. 2015;16(3):208. doi:10.1631/jzus.B1500017

6.	Watanabe Y, Chevalier B, Hayashida K, et al. Comparison of multislice computed tomography findings between bicuspid and tricuspid aortic valves before and after transcatheter aortic valve implantation. *Catheter Cardiovasc Interv*. 2015;86(2):323-330. doi:10.1002/ccd.25830

7.	Arai T, Lefèvre T, Hovasse T, et al. The feasibility of transcatheter aortic valve implantation using the Edwards SAPIEN 3 for patients with severe bicuspid aortic stenosis. *J Cardiol*. 2017;70(3):220-224. doi:10.1016/j.jjcc.2016.12.009

8.	Sannino A, Cedars A, Stoler RC, Szerlip M, Mack MJ, Grayburn PA. Comparison of Efficacy and Safety of Transcatheter Aortic Valve Implantation in Patients With Bicuspid Versus Tricuspid Aortic Valves. *Am J Cardiol*. 2017;120(9):1601-1606. doi:10.1016/j.amjcard.2017.07.053

9.	Yoon SH, Bleiziffer S, De Backer O, et al. Outcomes in Transcatheter Aortic Valve Replacement for Bicuspid Versus Tricuspid Aortic Valve Stenosis. *J Am Coll Cardiol*. 2017;69(21):2579-2589. doi:10.1016/j.jacc.2017.03.017

10.	Liao YB, Li YJ, Xiong TY, et al. Comparison of procedural, clinical and valve performance results of transcatheter aortic valve replacement in patients with bicuspid versus tricuspid aortic stenosis. *Int J Cardiol*. Published online 2018:69-74. Accessed November 13, 2024. https://dx.doi.org/10.1016/j.ijcard.2017.12.013

11.	Aalaei-Andabili SH, Beaver TM, Petersen JW, et al. Early and midterm outcomes of transcatheter aortic valve replacement in patients with bicuspid aortic valves. *J Card Surg*. 2018;33(9):489-496. doi:10.1111/jocs.13775

12.	De Biase C, Mastrokostopoulos A, Philippart R, et al. Aortic valve anatomy and outcomes after transcatheter aortic valve implantation in bicuspid aortic valves. *Int J Cardiol*. 2018;266:56-60. doi:10.1016/j.ijcard.2018.01.018

13.	Kim WK, Gaede L, Husser O, et al. Computed Tomography for Diagnosis and Classification of Bicuspid Aortic Valve Disease in Transcatheter Aortic Valve Replacement. *JACC Cardiovasc Imaging*. 2018;11(10):1539-1540. doi:10.1016/j.jcmg.2017.12.010

14.	Nagaraja V, Suh W, Fischman DL, et al. Transcatheter aortic valve replacement outcomes in bicuspid compared to trileaflet aortic valves. *Cardiovasc Revasc Med*. 2019;20(1):50-56. doi:10.1016/j.carrev.2018.09.013

15.	Xiong TY, Wang X, Li YJ, et al. Less pronounced reverse left ventricular remodeling in patients with bicuspid aortic stenosis treated with transcatheter aortic valve replacement compared to tricuspid aortic stenosis. *Int J Cardiovasc Imaging*. 2018;34(11):1761-1767. doi:10.1007/s10554-018-1401-6

16.	Mangieri A, Chieffo A, Kim WK, et al. Transcatheter aortic valve implantation using the ACURATE neo in bicuspid and tricuspid aortic valve stenosis: a propensity-matched analysis of a European experience. *EuroIntervention*. 2018;14(12):e1269-e1275. doi:10.4244/EIJ-D-18-00281

17.	Tchetche D, de Biase C, van Gils L, et al. Bicuspid Aortic Valve Anatomy and Relationship With Devices: The BAVARD Multicenter Registry. *Circ Cardiovasc Interv*. 2019;12(1):e007107. doi:10.1161/CIRCINTERVENTIONS.118.007107

18.	Pineda AM, Rymer J, Wang A, et al. Transcatheter aortic valve replacement for patients with severe bicuspid aortic stenosis. *Am Heart J*. 2020;224:105-112. doi:10.1016/j.ahj.2020.02.003

19.	Forrest JK, Kaple RK, Ramlawi B, et al. Transcatheter Aortic Valve Replacement in Bicuspid Versus Tricuspid Aortic Valves From the STS/ACC TVT Registry. *JACC: Cardiovascular Interventions*. 2020;13(15):1749-1759. doi:10.1016/j.jcin.2020.03.022

20.	Halim SA, Edwards FH, Dai D, et al. Outcomes of Transcatheter Aortic Valve Replacement in Patients With Bicuspid Aortic Valve Disease. *Circulation*. 2020;141(13):1071-1079. doi:10.1161/CIRCULATIONAHA.119.040333

21.	Kim WK, Bhumimuang K, Renker M, et al. Determinants of paravalvular leakage following transcatheter aortic valve replacement in patients with bicuspid and tricuspid aortic stenosis. *European Heart Journal - Cardiovascular Imaging*. 2021;22(12):1387-1396. doi:10.1093/ehjci/jeab011

22.	Makkar RR, Yoon SH, Chakravarty T, et al. Association Between Transcatheter Aortic Valve Replacement for Bicuspid vs Tricuspid Aortic Stenosis and Mortality or Stroke Among Patients at Low Surgical Risk. *JAMA*. 2021;326(11):1034-1044. doi:10.1001/jama.2021.13346

23.	Medranda GA, Rogers T, Forrestal BJ, et al. Balloon-Expandable Valve Geometry After Transcatheter Aortic Valve Replacement in Low-Risk Patients With Bicuspid Versus Tricuspid Aortic Stenosis. *Cardiovasc Revasc Med*. 2021;33:7-12. doi:10.1016/j.carrev.2021.03.027

24.	Xu Q, Liu X, Jiang J, et al. Transcatheter aortic valve replacement in atypical valve anatomy using the Lotus valve : A Chinese single-center experience. *Herz*. 2021;46(1):63-70. doi:10.1007/s00059-018-4778-z

25.	Jung JH, Kim HK, Park JB, et al. Progression of ascending aortopathy may not occur after transcatheter aortic valve replacement in severe bicuspid aortic stenosis. *Korean J Intern Med*. 2019;36(2):332-341. doi:10.3904/kjim.2019.089

26.	Deeb GM, Reardon MJ, Ramlawi B, et al. Propensity-Matched 1-Year Outcomes Following Transcatheter Aortic Valve Replacement in Low-Risk Bicuspid and Tricuspid Patients. *JACC: Cardiovascular Interventions*. 2022;15(5):511-522. doi:10.1016/j.jcin.2021.10.027

27.	Williams MR, Jilaihawi H, Makkar R, et al. The PARTNER 3 Bicuspid Registry for Transcatheter Aortic Valve Replacement in Low-Surgical-Risk Patients. *JACC: Cardiovascular Interventions*. 2022;15(5):523-532. doi:10.1016/j.jcin.2022.01.279

28.	Fan J, Chen J, Wang L, et al. Feasibility and safety of next-day discharge following transcatheter bicuspid aortic valve replacement. *IJC Heart & Vasculature*. 2022;42:101101. doi:10.1016/j.ijcha.2022.101101

29.	Jin Q, Chen S, Yang X, et al. Clinical outcomes of bicuspid versus tricuspid aortic valve stenosis after transcatheter aortic valve replacement with self-expandable valves. *BMC Cardiovascular Disorders*. 2022;22(1):540. doi:10.1186/s12872-022-02943-9

30.	Hong N, Pan W, Liu X, Zhou D, Wang J, Ge J. Transcatheter Aortic Valve Replacement for Bicuspid vs. Tricuspid Aortic Stenosis among Patients at Low Surgical Risk in China: From the Multicenter National NTCVR Database. *Journal of Clinical Medicine*. 2023;12(1):1. doi:10.3390/jcm12010387

31.	Mukai T, Kumamaru H, Kohsaka S, et al. One-year outcome of transcatheter aortic valve replacement for bicuspid aortic valve stenosis: a report from the Japanese Nationwide registry (J-TVT registry). *Cardiovasc Interv Ther*. 2023;38(4):414-423. doi:10.1007/s12928-023-00933-y

32.	Zheng HJ, Cheng YB, Lin DQ, et al. Effect of transcatheter aortic valve replacement on ascending aorta dilatation rate in patients with tricuspid and bicuspid aortic stenosis. *IJC Heart & Vasculature*. 2023;49:101313. doi:10.1016/j.ijcha.2023.101313

33.	Liu K, Wu K, Shen J, et al. Transcatheter aortic valve replacement in the treatment of bicuspid aortic stenosis with “down-size” interventional valves: procedural and mid-term follow-up. *Journal of Thoracic Disease*. 2024;16(1). doi:10.21037/jtd-23-1885

34.	Li H, Li W, Li J, et al. Long‐Term Durability of Transcatheter Aortic Valve Prostheses in Patients With Bicuspid Versus Tricuspid Aortic Valve. *Journal of the American Heart Association*. 2024;13(21):e035772. doi:10.1161/JAHA.124.035772

35.	Yamanaka F, Shishido K, Moriyama N, et al. Incidence and Prognosis of Prosthesis-Patient Mismatch After Transcatheter Aortic Valve Replacement for Bicuspid Aortic Stenosis. *JACC: Cardiovascular Interventions*. 2025;18(4):492-502. doi:10.1016/j.jcin.2024.10.002

36.	Zhu T, Luo J, Huang X, et al. Comparison of in-Hospital Complication Rates After Transcatheter Aortic Valve Replacement in Patients With Bicuspid Versus Tricuspid Aortic Valves: A Retrospective Cohort Study. *Catheterization and Cardiovascular Interventions*. 2025;106(7):3489-3497. doi:10.1002/ccd.70206

37.	Sheng W, Fan J, Chen J, et al. Transcatheter bicuspid versus tricuspid aortic valve replacement in patients with a small aortic annulus: an observational study. *Open Heart*. 2025;12(1). doi:10.1136/openhrt-2025-003357

38.	Louca A, Petursson P, Sundström J, et al. Comparative outcomes of transcatheter aortic valve replacement in bicuspid vs. tricuspid aortic valve stenosis patients: insights from the SWEDEHEART registry. *Int J Cardiol Heart Vasc*. 2025;59:101705. doi:10.1016/j.ijcha.2025.101705

39.	Sun Y, Wang C, Li J, et al. Outcomes of transcatheter aortic valve replacement in bicuspid versus tricuspid aortic stenosis with severe calcification. *Heart*. 2025;111(24):1207-1213. doi:10.1136/heartjnl-2024-324697

40.	Elbadawi A, Saad M, Elgendy IY, et al. Temporal Trends and Outcomes of Transcatheter Versus Surgical Aortic Valve Replacement for Bicuspid Aortic Valve Stenosis. *JACC: Cardiovascular Interventions*. 2019;12(18):1811-1822. doi:10.1016/j.jcin.2019.06.037

41.	Mentias A, Sarrazin MV, Desai MY, et al. Transcatheter Versus Surgical Aortic Valve Replacement in Patients With Bicuspid Aortic Valve Stenosis. *Journal of the American College of Cardiology*. 2020;75(19):2518-2519. doi:10.1016/j.jacc.2020.02.069

42.	Soud M, Al-khadra Y, Darmoch F, Pacha HM, Fanari Z, Alraies MC. Transcatheter aortic valve replacement in patients with bicuspid aortic valve stenosis: national trends and in-hospital outcomes. *Avicenna Journal of Medicine*. 2021;10:22-28. doi:10.4103/ajm.ajm_134_19

43.	Waksman R, Craig PE, Torguson R, et al. Transcatheter Aortic Valve Replacement in Low-Risk Patients With Symptomatic Severe Bicuspid Aortic Valve Stenosis. *JACC: Cardiovascular Interventions*. 2020;13(9):1019-1027. doi:10.1016/j.jcin.2020.02.008

44.	Husso A, Airaksinen J, Juvonen T, et al. Transcatheter and surgical aortic valve replacement in patients with bicuspid aortic valve. *Clin Res Cardiol*. 2021;110(3):429-439. doi:10.1007/s00392-020-01761-3

45.	Tsai HY, Lin YS, Wu IC, et al. Major adverse cardiac events and functional capacity in patients at intermediate risk undergoing transcatheter versus surgical aortic valve replacement for aortic stenosis with bicuspid valves. *J Card Surg*. 2021;36(3):828-833. doi:10.1111/jocs.15304

46.	Majmundar M, Kumar A, Doshi R, et al. Early outcomes of transcatheter versus surgical aortic valve implantation in patients with bicuspid aortic valve stenosis. *EuroIntervention*. 2022;18(1):23. doi:10.4244/EIJ-D-21-00757

47.	Sanaiha Y, Hadaya JE, Tran Z, Shemin RJ, Benharash P. Transcatheter and Surgical Aortic Valve Replacement in Patients With Bicuspid Aortic Valve Stenosis. *The Annals of Thoracic Surgery*. 2023;115(3):611-618. doi:10.1016/j.athoracsur.2022.06.030

48.	Ogami T, Yousef S, Brown JA, et al. Readmission-related outcomes of surgical versus transcatheter aortic valve replacement in patients aged 65 or older with bicuspid aortic valve. *Cardiovascular Revascularization Medicine*. 2024;63:16-20. doi:10.1016/j.carrev.2024.01.009

49.	Connolly JE, Herrmann HC, Atluri P, et al. Transcatheter aortic valve replacement achieves similar predicted effective orifice area to surgical aortic valve replacement in bicuspid aortic stenosis. *Cardiovascular Revascularization Medicine*. 2024;66:21-26. doi:10.1016/j.carrev.2024.03.017

50.	Kassab J, Desai P, Keshvani N, et al. Transcatheter vs. surgical aortic valve replacement in bicuspid aortic valve stenosis. *Eur Heart J Open*. 2025;5(5):oeaf110. doi:10.1093/ehjopen/oeaf110

51.	Jørgensen TH, Savontaus M, Willemen Y, et al. Three-Year Follow-Up of the NOTION-2 Trial: TAVR Versus SAVR to Treat Younger Low-Risk Patients With Tricuspid or Bicuspid Aortic Stenosis. *Circulation*. 2025;152(19):1326-1337. doi:10.1161/CIRCULATIONAHA.125.076678

52.	Mehaffey JH, Jagadeesan V, Kawsara M, et al. Transcatheter vs Surgical Aortic Valve Replacement in Bicuspid Aortic Valves. *Ann Thorac Surg*. 2025;119(6):1261-1269. doi:10.1016/j.athoracsur.2024.11.023
